# Selection Estimation from Genetic Time-Series Data: Effects of Limited Sampling and Genetic Drift

**DOI:** 10.1093/molbev/msaf301

**Published:** 2025-11-28

**Authors:** Qingbei Cheng, Muhammad Saqib Sohail, Matthew R McKay

**Affiliations:** Department of Electronic and Computer Engineering, Hong Kong University of Science and Technology, Hong Kong SAR, China; Department of Computer Science, Bahria University, Lahore, Pakistan; Department of Electrical and Electronic Engineering, University of Melbourne, Melbourne, VIC, Australia; Department of Microbiology and Immunology, The Peter Doherty Institute for Infection and Immunity, University of Melbourne, Melbourne, VIC, Australia

**Keywords:** limited sampling, genetic drift, time-series, selection, inference

## Abstract

Estimating selection from genetic time-series data is fundamental to understanding evolutionary dynamics. Accurate selection inference is confounded by multiple noise sources, including limited sampling of populations and genetic drift. To characterize how these uncertainties collectively affect estimator performance, we analyze a mathematically tractable selection coefficient estimator derived under the marginal path likelihood (MPL) framework. We identify a parameter, the integrated mutant allele variance, as a key quantity determining estimator precision. Our analysis reveals that variance integration mitigates sampling and genetic drift errors at different rates, with drift typically becoming the dominant source of error in longer trajectories. The increased robustness of MPL-based estimation to sampling is surprising, since MPL is derived from a model that neglects this effect. Our findings offer insights into how incorporating temporal information reduces multiple sources of noise when estimating selection coefficients.

## Introduction

Selection inference is a fundamental topic in population genetics. Quantifying selection is essential for understanding how species adapt to environmental pressures (Bollback and Huelsenbeck 2007; Barrick et al. 2009; Ludwig et al. 2009; Orozco-Terwengel et al. 2012; Burke et al. 2014; Henn et al. 2015). Accurate interpretation of selection plays a critical role in various practical applications, such as studies on drug resistance (Renzette et al. 2014; Feder et al. 2016; Zhang et al. 2023), vaccine design (Quadeer et al. 2019; Gu et al. 2023; Rouzine and Rozhnova 2023), genetic variations (Feder et al. 2014; Zanini et al. 2017; Connallon and Chenoweth 2019; Ito et al. 2021), and cancer evolution (Rozhok and DeGregori 2015; Williams et al. 2016; Łuksza et al. 2017; Salehi et al. 2021).

**Fig. 1.**
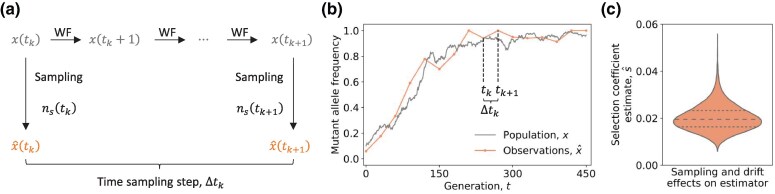
Illustration of limited sampling and genetic drift effects analyzed in this study. a) Key notations are introduced, and the observation of mutant allele frequencies across two consecutively sampled generations, $t_{k}$ and $t_{k+1}$, is depicted. During this interval, the population mutant allele frequency evolves under the Wright–Fisher (WF) model with effects of genetic drift, selection, and mutation. The observed mutant allele frequencies, $\hat{x}(t_{k})$ and $\hat{x}(t_{k+1})$, are estimated from incomplete sampling of the population frequencies, $x(t_{k})$ and $x(t_{k+1})$. The time sampling step $\Delta t_{k}=t_{k+1}-t_{k}$ accounts for the missing temporal information between generations $t_{k}$ and $t_{k+1}$. b) An example trajectory of the population mutant allele frequency, alongside a corresponding observed trajectory is presented. c) The empirical distribution of a selection coefficient estimator obtained from multiple observed mutant allele frequency trajectories under joint effect of limited sampling and genetic drift is displayed.

Time-series datasets sequenced from evolving populations offer valuable opportunities to examine the mechanisms of evolution in detail. Compared to selection coefficient estimates obtained from datasets with only one or two time points, estimates from genetic time-series with additional time points can yield more reliable results (Buffalo and Coop 2019; Lynch and Ho 2020). Nevertheless, estimating selection coefficients from time-series data is confounded by effects of limited (incomplete) population sampling and genetic drift (Fig. 1). Limited sampling introduces noise into observed mutant allele frequencies, while genetic drift leads to stochastic changes in allele frequencies due to a finite population size. The relative effects of limited sampling and genetic drift on the performance of time-series selection coefficient estimators are currently not well understood.

Selection coefficient estimators that account for both limited sampling and genetic drift are often based on hidden Markov models (HMMs), where transition probabilities reflect the population evolutionary dynamics and emission probabilities capture the sampling effect (Bollback et al. 2008; Malaspinas et al. 2012; Mathieson and McVean 2013; Lacerda and Seoighe 2014; Steinrücken et al. 2014; Vitalis et al. 2014; Ferrer-Admetlla et al. 2016; Gompert 2016; Iranmehr et al. 2017; He et al. 2020; Vaughn and Nielsen 2024; Fine and Steinrücken 2025). These HMM-based methods lead to complex likelihood functions that are analytically intractable. Approaches based on approximate Bayesian computation circumvent the need for explicit likelihood calculations by approximating the posterior distribution through Monte Carlo simulations (Beaumont 2010; Peter et al. 2012; Foll et al. 2014, 2015; Sackman et al. 2019; Zinger et al. 2019). Moreover, recent methods have employed neural networks to infer selection coefficients using statistical features extracted from evolutionary histories (Hejase et al. 2022; Whitehouse and Schrider 2023). All these approaches rely on numerical optimization techniques and lack closed-form solutions. Therefore, it is difficult to conduct analytical studies of their performance. Simplified approaches have also been proposed that model limited sampling but not genetic drift, fitting observed mutant allele frequency trajectories to deterministic curves (Illingworth and Mustonen 2011; Illingworth et al. 2012, 2014; Jewett et al. 2016; Taus et al. 2017; Lynch and Ho 2020). These methods often require numerical evaluation and can be challenging to interpret, except for a few that apply least-squares regression on logit-transformed frequency trajectories (Taus et al. 2017; Lynch and Ho 2020). For these estimators, ignoring the contribution of genetic drift can lead to an inaccurate evaluation of estimator performance, which becomes significant as the effect of genetic drift accumulates over time (Lynch and Ho 2020).

Recently, we introduced a novel marginal path likelihood (MPL) framework for selection coefficient estimation that is mathematically tractable, interpretable, and shown to be accurate over a wide range of evolutionary conditions (Sohail et al. 2021). A detailed performance comparison of the MPL-based selection coefficient estimator with several other approaches in literature is provided in Sohail et al. (2021). The key to the MPL-based approach is that it provides tractable likelihood formulas by employing tools from statistical physics. The approach explicitly models genetic drift but not limited sampling, which technically is best justified when the evolving population is well sampled (Valleriani 2016; Liu et al. 2019). Despite this assumption, our simulation studies suggested that the MPL-based approach is robust even under highly limited sampling conditions. However, a basic statistical understanding of the performance of MPL-based estimators and their dependence on different evolutionary parameters has not yet been established. While many works have introduced selection coefficient estimators under different modeling approaches, comparatively fewer have focused on analytically studying their performance (Lynch and Ho 2020; Sant et al. 2022; Jenkins 2024, unpublished data).

The key contribution of this work is the performance evaluation of MPL-based selection coefficient estimators through a combination of analytical and empirical results. Focusing on single-locus models, we quantify the estimator performance under limited sampling and genetic drift effects, determining the key evolutionary factors that influence its precision. Our results provide insights into the robustness of MPL-based estimators with respect to confounding effects of limited sampling and genetic drift, highlighting the relative contribution of these noise components to the overall estimator performance. Our analysis is enabled by a mathematical decomposition that we present, which allows the two noise components to be studied separately. Our results, in general, provide novel insights into the relative effects of limited sampling and genetic drift in time-series-based inference of selection, and highlight the importance of utilizing longer temporal trajectories for achieving accurate estimation performance.

## Results

### Selection Coefficient Estimator

We investigate a single-locus selection coefficient estimator derived by considering a population evolving under a stochastic Wright–Fisher (WF) model with selection and mutation, and following the MPL framework that we introduced earlier (Sohail et al. 2021, 2022). This framework models the stochastic effect of genetic drift of an evolving population but does not model the effect of limited sampling. MPL uses a diffusion approximation of the WF process and a path integral approach from statistical physics to efficiently represent the probability of the population mutant allele frequency following a particular trajectory (Materials and Methods). More specifically, for a population mutant allele frequency trajectory observed at *K* nonconsecutive generations $\left(t_{1},t_{2},\ldots ,t_{K}\right)$, MPL expresses the probability of obtaining a population mutant allele frequency trajectory $\left(x(t_{1}),x(t_{2}),\ldots ,x(t_{K})\right)$, given the initial mutant allele frequency $x(t_{0})$, as


(1) $$P\left({\left(x(t_{k})\right)}_{k=1}^{K}\;|\;x(t_{0}),N,\mu ,s\right)=\prod_{k=0}^{K-1}P\left(x(t_{k+1})\;|\;x(t_{k}),N,\mu ,s\right),$$

where *N* is the finite population size, *μ* is the mutation probability, and *s* is the selection coefficient of the mutant allele. The transition probability $P(x(t_{k+1})\;|\;x(t_{k}),N,\mu ,s)$ denotes the probability of observing a population mutant allele frequency of $x(t_{k+1})$ at generation $t_{k+1}$, given the population mutant allele frequency $x(t_{k})$ at generation $t_{k}$.

The MPL framework (Sohail et al. 2021, 2022) approximates the probability in (1) using the path integral approach (Risken 1989). This procedure consists of two steps. First, the WF process is approximated as a diffusion process (Kimura 1964; Ewens 2004; Durrett 2008; Bollback et al. 2008; Malaspinas et al. 2012; Steinrücken et al. 2014; Tataru et al. 2015; Ferrer-Admetlla et al. 2016; He et al. 2017; Sohail et al. 2021). This allows the transition probabilities in (1) to be expressed in the form of transition probability density of a diffusion process (Durrett 2008). Next, the transition probability density is discretized over small time intervals resulting in a Gaussian form (Materials and Methods); allowing for an analytical expression of the selection coefficient under the maximum likelihood criterion, i.e.


(2) $${\hat{s}}_{\mathrm{MPL}}=\underset{s}{\arg\;\max}\;L(s\;|\;{\left(x(t_{k})\right)}_{k=0}^{K},N,\mu ),$$

where $L(s\;|\;(x(t_{k}){)}_{k=0}^{K},N,\mu )=P((x(t_{k}){)}_{k=1}^{K}\;|\;x(t_{0}),N,\mu ,s)$. Solving (2) leads to a closed-form solution (Materials and Methods):


(3) $${\hat{s}}_{\mathrm{MPL}}=\frac{x(t_{K})-x(t_{0})-\mu \sum_{k=0}^{K-1}\Delta t_{k}\left(1-2x(t_{k})\right)}{\sum_{k=0}^{K-1}\Delta t_{k}v(t_{k})},$$

where $\Delta t_{k}=t_{k+1}-t_{k}$ is the time sampling step between generations $t_{k}$ and $t_{k+1}$, and $v(t_{k})=x(t_{k})(1-x(t_{k}))$ is the population mutant allele variance at generation $t_{k}$. The derivation of (3) assumes that both the selection coefficient *s* and the mutation probability *μ* are of $O(N^{-1})$, where *N* is the population size (Materials and Methods). Intuitively, the numerator in (3) represents the net mutant allele frequency change along the trajectory, adjusted for changes in frequency due to mutation, while the denominator represents the integrated variance along the trajectory.

In practice, the mutant allele frequencies in (3) are unobservable and are estimated by sampling the population. Under a standard binomial sampling model, with $n_{s}(t_{k})$ samples drawn at generation $t_{k}$, the estimated frequency $\hat{x}(t_{k})$ follows


(4) $$n_{s}(t_{k})\hat{x}(t_{k})∼\mathrm{Bin}(n_{s}(t_{k}),x(t_{k})),$$

which may be substituted into (3). This substitution however introduces a statistical bias in the mutant allele variance, $\hat{v}(t_{k})=\hat{x}(t_{k})(1-\hat{x}(t_{k}))$. The bias can be corrected with the following adjustment (Materials and Methods):


(5) $$\hat{s}=\frac{\hat{x}(t_{K})-\hat{x}(t_{0})-\mu \sum_{k=0}^{K-1}\Delta t_{k}\left(1-2\hat{x}(t_{k})\right)}{\sum_{k=0}^{K-1}\left(\frac{n_{s}(t_{k})}{n_{s}(t_{k})-1}\right)\Delta t_{k}\hat{v}(t_{k})}.$$

We pursued analytical and empirical analysis to statistically characterize the performance of the estimator $\hat{s}$ and to quantify the effects (and relative effects) of the two primary sources of errors: limited sampling and genetic drift.

### Estimator Performance under Limited Sampling

We first assessed the effect of limited sampling errors on the performance of the estimator $\hat{s}$ (5), considering a model that is void of genetic drift. This is done by focusing on a population evolving under a deterministic evolutionary model. The deterministic evolutionary model is analogous to the WF model, assuming an infinite population size that eliminates the stochastic fluctuations due to genetic drift.

**Fig. 2.**
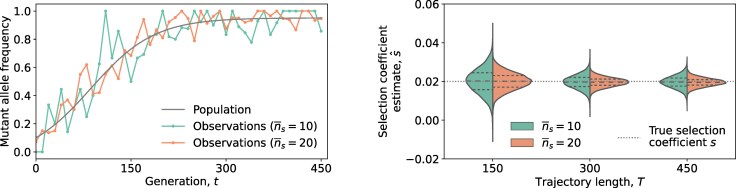
Variance of estimator $\hat{s}$ (5) reduces as the sample size or the trajectory length increases. The *left* panel shows a population mutant allele frequency trajectory simulated under a deterministic evolutionary model, and examples of its observations with time-varying sample sizes. At each time point, the sample size is a Poisson random variable with a mean value of ${\bar{n}}_{s}=(10,20)$, respectively. The *right* panel shows empirical distributions of the selection coefficient estimates $\hat{s}$ obtained from $10^{4}$ observed mutant allele frequency trajectories with trajectory length T=(150,300,450). Simulation parameters were selection coefficient s=0.02, initial mutant allele frequency x(0)=0.1, mutation probability $\mu =10^{-3}$, with a constant time sampling step $\Delta t_{k}=10$ for k=(0,1,…,K−1).

Under the deterministic evolutionary model, our simulations revealed that the estimator $\hat{s}$ had a mean very close to the true selection coefficient *s*, while the variance was reduced when either the expected sample size ${\bar{n}}_{s}$ or the trajectory length (i.e. the number of generations used for inference) *T* increased (Fig. 2). Moreover, the correction factor for the statistical bias in $\hat{v}(t_{k})$ employed in (5) improved the estimator performance under limited sampling conditions by centering the empirical distribution of the estimates around the true selection coefficient (Supplementary Fig. S1). Notably, the estimator variance could be small even if very limited samples were available per sampling time point (i.e. ${\bar{n}}_{s}=10$), provided that the temporal trajectory was sufficiently long (larger *T*). This suggests that integrating temporal measurements can compensate for errors in estimated selection coefficients due to limited sampling. Simulations further suggested that the probability distribution of $\hat{s}$ tended to a Gaussian shape as either ${\bar{n}}_{s}$ or *T* increased (*right* panel of Fig. 2).

Mathematical analysis was conducted to gain further insights into these observed trends, for the estimator mean, variance, and distributional properties.

#### Estimator Mean and Variance

The analytical form of (5) enables the statistical properties of $\hat{s}$ to be characterized mathematically and to quantify more precisely how the estimator performance depends on the sample size and the trajectory length. For convenience and analytical tractability, we assume a constant sample size $n_{s}$ and a constant time sampling step Δt. Later, simulations show that our results hold even if the sample size is time-varying.

Adopting an approach based on the multivariate Taylor series expansion about the mean of the numerator and denominator in (5) and retaining the first-order terms, we derived analytical expressions for the mean and variance (see Materials and Methods). For the mean, this led to


(6) $$\text{E}[\hat{s}]={\hat{s}}_{\mathrm{MPL}},$$

where ${\hat{s}}_{\mathrm{MPL}}$ defined in (3) corresponds to the (unobservable) estimator with perfect population sampling. Moreover, under the deterministic evolutionary model,


(7) $${\hat{s}}_{\mathrm{MPL}}=s,$$

for sufficiently small *s* and *μ* (see Materials and Methods). This confirms that, under the assumed modeling conditions, the estimator $\hat{s}$ (5) is unbiased.

For the variance, our analysis produced an explicit formula (see Materials and Methods), revealing fluctuations going to zero with $n_{s}$ and *V* at a rate of


(8) $$\text{Var}[\hat{s}]=O(n_{s}^{-1}V^{-2}),$$

where


(9) $$V=\Delta t\sum_{k=0}^{K-1}v(t_{k})=\Delta t\sum_{k=0}^{K-1}x(t_{k})\left(1-x(t_{k})\right)$$

is the integrated variance of the mutant allele frequency trajectory computed over T=KΔt generations.

Importantly, the scale of the estimator variance (8) is determined by the product of $n_{s}$ and $V^{2}$, reflecting that for a given $n_{s}$, sampling over longer trajectories improves the precision of the estimator. However, the trajectory length *T* itself is not the primary factor; rather it is the mutant allele variance integrated along the trajectory, *V*, that is important. The integrated variance monotonically increases with the trajectory length whenever the trajectory is away from the boundary ($x(t_{k})$ is not 0 or 1), with time points where $x(t_{k})$ is near 0.5 having the greatest impact.

**Fig. 3.**
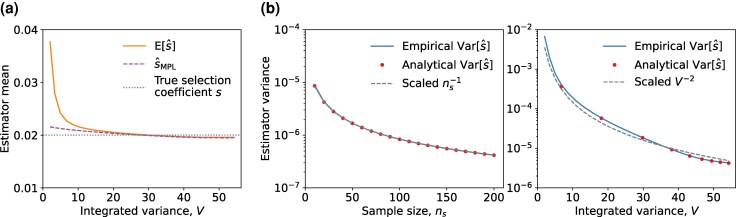
Our analytical expressions for the estimator mean (6) and variance (8) accurately reflect the empirical results under the deterministic evolutionary model. a) The empirical estimator mean E[$\hat{s}$] given a fixed sample size ($n_{s}=20$), the estimate ${\hat{s}}_{\mathrm{MPL}}$ (3) obtained from the population mutant allele frequency trajectory without limited sampling effect, along with the true selection coefficient *s* are shown. b) The estimator variance Var[$\hat{s}$] is of order $O(n_{s}^{-1}V^{-2})$ is shown. In the *left* panel, we fixed the trajectory length T=450 (thus fixed the integrated variance *V*) and plotted the empirical estimator variance with respect to sample sizes $n_{s}$ between 10 and 200. Values of the analytical estimator variance were obtained following (34) presented in Materials and Methods. In the *right* panel, we fixed $n_{s}=20$ and plotted the empirical estimator variance with respect to integrated variance values *V* by varying *T*. Values of the analytical estimator variance were plotted when T=(50,100,…,450). The factors of $n_{s}^{-1}$ and $V^{-2}$ were scaled by constants obtained from fitting the empirical estimator variance with a least-squares loss function. Simulation parameters were selection coefficient s=0.02, initial mutant allele frequency x(0)=0.1, mutation probability $\mu =10^{-3}$, with time sampling step Δt=10. Empirical values of the estimator mean and variance were obtained from $10^{6}$ observations of the population mutant allele frequency trajectory.

While the analytical expressions in (6), (7), and (8) are based on asymptotic expansions, simulations revealed that they accurately approximated the estimator mean (Fig. 3a) and variance (Fig. 3b). They also remained accurate for different parameter settings, such as for different initial mutant allele frequencies (Supplementary Fig. S2) and selection coefficients (Supplementary Fig. S3), although with a slight downward bias observed for large selection coefficients. Relaxing the assumption of constant sample size by allowing it to be a time-varying Poisson random variable retains the insights discussed previously. Simulations showed that the mean of $\hat{s}$ was close to both ${\hat{s}}_{\mathrm{MPL}}$ and the true selection coefficient *s* (Supplementary Fig. S4A), and the variance of $\hat{s}$ scaled as $O({\bar{n}}_{s}^{-1}V^{-2})$, where ${\bar{n}}_{s}$ represented the average sample size (Supplementary Fig. S4B).

#### Estimator Distribution

Further analysis demonstrated that estimator $\hat{s}$ (5) follows a Gaussian distribution for sufficiently large integrated variance *V* (correspondingly, for sufficiently long polymorphic trajectories). This result was obtained by leveraging the central limit theorem and a classical statistical result on the ratio of Gaussian variables (Hinkley 1969), assuming the sample size $n_{s}$ is sufficiently large (see Materials and Methods for details).

**Fig. 4.**
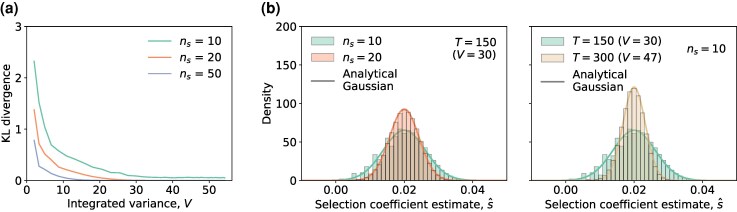
The distribution of $\hat{s}$ (5) converges to a Gaussian distribution as the sample size or the integrated variance increases. a) shows the Kullback–Leibler (KL) divergence between the standardized empirical distribution of $\hat{s}$ (i.e. normalized to zero-mean and unit-variance) and the reference Gaussian distribution N(0,1) under various sample sizes $n_{s}=(10,20,50)$. The *left* panel of b) shows the distribution of $\hat{s}$ for different sample sizes $n_{s}$ and fixed trajectory length *T* and thus a fixed integrated variance *V*. The *right* panel of b) shows the same for a fixed sample size $n_{s}$ and different trajectory length *T* (and thus different integrated variance *V*). The mean of the analytical Gaussian probability densities (drawn with a solid line) in b) was the true selection coefficient *s*, while the variance was obtained from (34) presented in Materials and Methods. The empirical distributions of $\hat{s}$ were obtained from $10^{6}$ observations of the population mutant allele frequency trajectory simulated under the deterministic evolutionary model over 450 generations. Simulation parameters were selection coefficient s=0.02, initial mutant allele frequency x(0)=0.1, mutation probability $\mu =10^{-3}$, with time sampling step Δt=10.

Simulations showed that convergence occurred rapidly (Fig. 4a), demonstrating that the Gaussian approximation was accurate even for small sample sizes and small integrated variances. Hence, with the mean and variance expressions (presented in Materials and Methods), a closed-form approximation for the distribution of $\hat{s}$ (5) can be computed directly from the population mutant allele frequency trajectory (Fig. 4b). This distribution result can be used for efficiently computing estimator statistics, such as the probability of correctly classifying a beneficial mutation under limited sampling effect, without requiring extensive numerical simulations.

### Estimator Performance under Joint Effect of Limited Sampling and Genetic Drift

In practice, genetic drift presents a significant evolutionary force that confounds the inference of selection. Fluctuations in allele frequencies due to drift are cumulative across a trajectory (unlike fluctuations due to limited sampling), which makes quantifying the effect of drift on estimator performance challenging. Here, we present a semi-analytical approach that extends our analysis to include the effect of genetic drift in addition to limited sampling. This analysis enables the relative contributions of these two noise sources on performance to be evaluated and compared.

#### Estimator Mean

Under the joint effect of limited sampling and genetic drift, the mean of estimator $\hat{s}$ is given by (Materials and Methods)


(10) $$\begin{array}{rl} \text{E}[\hat{s}] & ={\text{E}}_{d}\left[{\text{E}}_{s}\left[\hat{s}\;|\;(x(t_{k}){)}_{k=0}^{K}\right]\right]\\ & ={\text{E}}_{d}\left[{\hat{s}}_{\mathrm{MPL}}\right]\\ & =s. \end{array}$$

Here, ${\text{E}}_{s}[\hat{s}\;|\;(x(t_{k}){)}_{k=0}^{K}]$ denotes the estimator mean given a realization of the WF trajectory $\left(x(t_{0}),x(t_{1}),\ldots ,x(t_{K})\right)$, averaged with respect to fluctuations due to limited sampling; while ${\text{E}}_{d}$ represents the mean over WF trajectories (i.e. capturing fluctuations due to genetic drift). The first step in (10) follows under the law of total expectation. For a given WF trajectory $(x(t_{k}){)}_{k=0}^{K}$, the estimator mean under limited sampling ${\text{E}}_{s}[\hat{s}\;|\;(x(t_{k}){)}_{k=0}^{K}]$ is written as ${\hat{s}}_{\mathrm{MPL}}$ following similar arguments as used to obtain (6). The second step in (10) follows via a first-order multivariate Taylor series expansion of the expression of ${\hat{s}}_{\mathrm{MPL}}$ (see Materials and Methods for details).

**Fig. 5.**
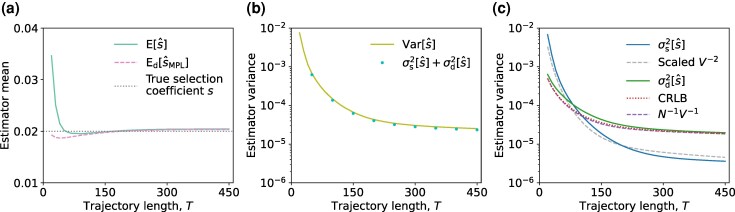
Derived analytical expressions for the estimator mean and variance accurately reflect the empirical results under the stochastic evolutionary model. a) The empirical mean of $\hat{s}$ (5) under the joint effect of limited sampling and genetic drift, as well as the empirical mean of ${\hat{s}}_{\mathrm{MPL}}$ (3) under genetic drift effect are shown. b) The empirical variance of $\hat{s}$ under the joint effect of limited sampling and genetic drift, as well as the empirical sum of sampling-only variance $\sigma_{s}^{2}[\hat{s}]$ and drift-only variance $\sigma_{d}^{2}[\hat{s}]$ when T=(50,100,…,450) are shown. c) The sampling-only variance $\sigma_{s}^{2}[\hat{s}]$ is of order $O(V^{-2})$, while the drift-only variance $\sigma_{d}^{2}[\hat{s}]$ is approximately 1/NV (close to its CRLB) is shown. Empirical values of estimator mean and variance were obtained from $10^{5}$ trajectories simulated over 450 generations. Simulation parameters were population size N=1,000, selection coefficient s=0.02, initial mutant allele frequency x(0)=0.1, mutation probability $\mu =10^{-3}$, with sample size $n_{s}=20$ and time sampling step Δt=10.

The result in (10) indicates that the estimator $\hat{s}$ remains unbiased (up to a first order approximation), even in the presence of genetic drift. Our simulations validated this result (Fig. 5a), including for a range of parameter settings (Supplementary Figs. S5A, S6A, and S7A).

#### Estimator Variance

The variance of $\hat{s}$, Var[$\hat{s}$], is difficult to analyze when considering both limited sampling and genetic drift effects. However, extensive simulations indicated that this variance could be accurately approximated (Fig. 5b) by the decomposition


(11) $$\text{Var}[\hat{s}]\approx \sigma_{s}^{2}[\hat{s}]+\sigma_{d}^{2}[\hat{s}],$$

where we refer to the first term $\sigma_{s}^{2}[\hat{s}]$ as the sampling-only variance, and the second term $\sigma_{d}^{2}[\hat{s}]$ as the drift-only variance. The sampling-only variance quantifies the effect of limited sampling on the estimator variance, for a given population mutant allele frequency trajectory (without considering genetic drift). This component is derived from estimates obtained from noisy observations of the mean population mutant allele frequency trajectory, which is calculated by averaging across trajectories simulated under the WF model. In contrast, the drift-only variance reflects the impact of genetic drift on the estimator variance. This component is derived from estimates obtained from realizations of population mutant allele frequency trajectories under the WF model (without limited sampling effect). This decomposition (11) was validated as accurate and robust across a range of parameter settings (Supplementary Figs. S5B, S6B, and S7B).

These results indicate that the fluctuations due to limited sampling and genetic drift may be treated as uncorrelated. This simplifies statistical analysis, since the effects of limited sampling and genetic drift can be studied separately. For the sampling-only variance $\sigma_{s}^{2}[\hat{s}]$, similar to (8), we have


(12) $$\sigma_{s}^{2}[\hat{s}]=O(n_{s}^{-1}V^{-2}).$$

This is because the mean trajectory under the WF evolutionary model closely follows the population mutant allele frequency trajectory under the deterministic evolutionary model (Supplementary Fig. S8). The result in (12) was validated via simulations (Fig. 5c), and for various parameter settings (Supplementary Figs. S5C, S6C, and S7C).

Theoretical computation of the drift-only variance $\sigma_{d}^{2}[\hat{s}]$ is more challenging. Instead, we derived a Cramér-Rao Lower Bound (CRLB), representing the minimum variance that the estimator can achieve (Kay 1993). For an unbiased estimator of parameter *s*, the CRLB is expressed as the inverse of the Fisher information I(s). Our analysis showed that the estimator $\hat{s}$ can be considered unbiased for a WF model with perfect sampling (see Materials and Methods), and I(s) can be computed explicitly. This leads to a simple lower bound on the drift-only variance (see Materials and Methods for details):


(13) $$\sigma_{d}^{2}[\hat{s}]\geq I^{-1}(s)=\frac{1}{NV}.$$

Here, *N* is the population size and *V* is the integrated variance under the deterministic evolutionary model defined in (9). Our simulations showed that this lower bound was tight, particularly when *N* was not small (Fig. 5c, Supplementary Figs. S5C, S6C, and S7C).

The expressions (11)–(13) for the estimator variance further provide insight into the relative effects of limited sampling and genetic drift on estimator performance. Most notably, for given population and sample sizes, they show that temporal sampling of polymorphic allele frequency trajectories (reflected through the integrated variance *V*) has the dual effect of mitigating both sources of errors. Moreover, the mitigation of errors due to limited sampling is particularly significant, with the sampling-only variance $\sigma_{s}^{2}[\hat{s}]$ decreasing rapidly as $O(V^{-2})$. The drift-only variance decreases as $O(V^{-1})$, suggesting that with sufficient temporal sampling of polymorphic mutant allele frequency trajectories, the estimator performance may be dominated by fluctuations due to genetic drift and not limited sampling. The leading factors of $O(n_{s}^{-1})$ and $O(N^{-1})$ in (11) and (13) also control for the effects of limited sampling and genetic drift fluctuations, respectively, suggesting that the impact of limited sample size per time point depends on the population size. Despite this, our simulations demonstrated that genetic drift effect was dominant for conditions even when just 1% of the population was sampled (Supplementary Fig. S9), and for population sizes as large as N=50,000.

#### Effect of Temporal Sampling Resolution

**Fig. 6.**
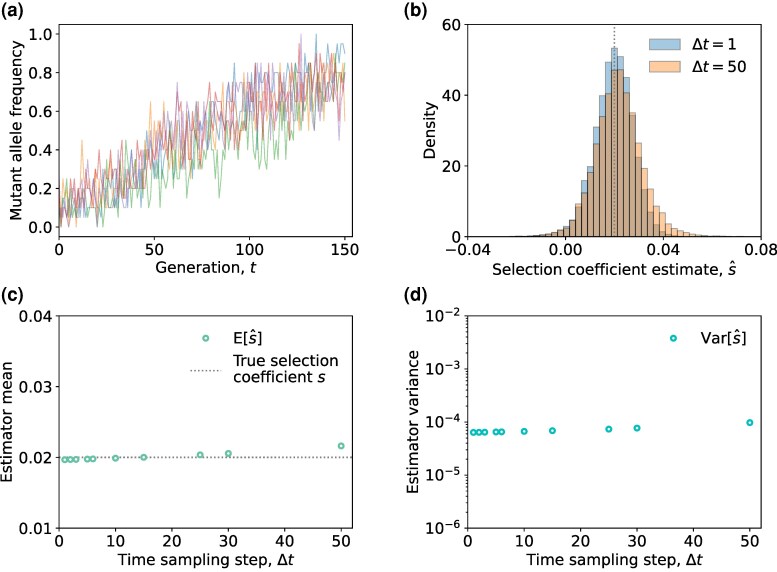
Selection coefficient estimator $\hat{s}$ demonstrates similar performance across a range of time sampling steps. a) Examples of observed mutant allele frequencies under the joint effect of limited sampling and genetic drift, where most observed frequencies are polymorphic are shown. b) The empirical distributions of $\hat{s}$ (5) with time sampling steps Δt=(1,50), where the dotted line indicates the true selection coefficient *s* are shown. c) The empirical mean of $\hat{s}$ with respect to the time sampling step Δt, along with the true selection coefficient *s* is shown. d) The empirical variance of $\hat{s}$ under the joint effect of limited sampling and genetic drift with respect to the time sampling step Δt is shown. Empirical values of estimator mean and variance were obtained from $10^{5}$ trajectories simulated over 150 generations. Simulation parameters were population size N=1,000, selection coefficient s=0.02, initial mutant allele frequency x(0)=0.1, mutation probability $\mu =10^{-3}$, with sample size $n_{s}=20$. The time sampling step was set to Δt=(1,2,3,5,6,10,15,25,30,50), i.e. divisors of the trajectory length *T*.

Next, we studied the impact of varying the time sampling step Δt on the performance of estimator $\hat{s}$ (5). We considered a scenario where the population mutant allele frequency was polymorphic at most time points (Fig. 6a), such that reducing the time sampling step resulted in more observed time points with mutant allele frequencies that were away from the boundaries. For a fixed trajectory length *T*, the estimate obtained from four observed time points (Δt=50) was comparable to that obtained from observations taken at every generation (Δt=1) (Fig. 6b). This robustness was reflected in the mean and variance across a range of Δt values (Fig. 6c,d). These results, alongside our study of the estimator mean and variance with varying trajectory lengths (Fig. 5), suggest that obtaining additional intermediate time points does not significantly enhance estimator precision. Instead, extending trajectory length to include informative time points where mutant allele frequencies are away from the boundaries is more effective. These findings are consistent with those reported in the literature (Schraiber et al. 2013; Topa et al. 2015; Taus et al. 2017; Lynch and Ho 2020; Phillips et al. 2020).

Overall, our results in this section highlight the robustness of MPL-based time-series selection estimation to limited sampling and genetic drift effects. They demonstrate the importance of sampling populations temporally to capture high integrated variance *V*, and provide insight into the relative effects of limited sampling and genetic drift in determining estimator performance.

### Comparison with Alternative Time-Series Estimation Approaches That Model Limited Sampling

The MPL-based estimator $\hat{s}$ (5) we have analyzed is derived under a model that accounts for genetic drift but not limited sampling effect. Despite these modeling assumptions, the estimator achieves robust performance against both drift and sampling. As a performance benchmark, here we investigate how $\hat{s}$ performs compared with estimators derived under models that account for both limited sampling and genetic drift effects. As indicated earlier, such estimators involve more complicated likelihood functions, requiring numerical methods to evaluate, and are not analytically tractable.

**Fig. 7.**
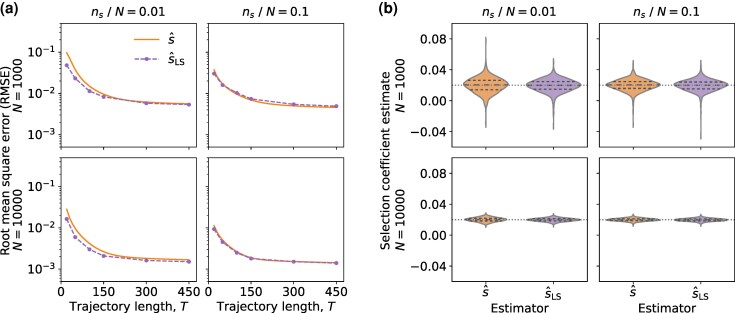
The performance of estimators $\hat{s}$ and ${\hat{s}}_{\mathrm{LS}}$ becomes increasingly similar as the trajectory length *T* increases. a) The root mean square error (RMSE) between the estimates and the true selection coefficient *s* is shown. b) The distributions of $\hat{s}$ and ${\hat{s}}_{\mathrm{LS}}$ with T=150 generations used for inference, where the dotted lines indicate the true selection coefficient *s* are shown. Results were empirically obtained from 1,000 trajectories, with various population and sample sizes as indicated. Simulation parameters were selection coefficient s=0.02, initial mutant allele frequency x(0)=0.1, mutation probability $\mu =10^{-3}$, with time sampling step Δt=10.

We conducted the comparison using simulated data in which all parameters were known. To enable a direct comparison with the MPL-based estimator $\hat{s}$, we extended the MPL framework to incorporate limited sampling effect. This led to a HMM formulation, with the resulting estimator denoted ${\hat{s}}_{\mathrm{LS}}$. Specifically, the transition probabilities of the HMM were computed using the same approach as that employed in the MPL framework, and emission probabilities modeled the binomial sampling process (see Materials and Methods for details). Simulations demonstrated that both estimators $\hat{s}$ and ${\hat{s}}_{\mathrm{LS}}$ had similar performance. The HMM estimator ${\hat{s}}_{\mathrm{LS}}$ performed slightly better for short trajectories and with very limited sampling ($n_{s}/N=0.01$), however, any such difference became negligible as the trajectory length increased (Fig. 7a). The empirical distributions of $\hat{s}$ and ${\hat{s}}_{\mathrm{LS}}$ were also found to be comparable (Fig. 7b). Additional simulations confirmed the similar performance of the estimators $\hat{s}$ and ${\hat{s}}_{\mathrm{LS}}$ across a wide range of population sizes and sampling conditions (Supplementary Figs. S10 and S11).

We also compared the performance of the MPL-based estimator $\hat{s}$ with established approaches derived from HMMs that account for both limited sampling and genetic drift effects. We used a classic real-world *medionigra* morph dataset (Materials and Methods), where the true parameters remain unknown. Applying the MPL-based approach to this dataset returned selection coefficient estimates that matched closely with the benchmark results reported in the literature (Table 1). Consistent with findings from the comparisons using simulated data, the MPL-based estimator demonstrated similar performance to these more complex estimators on real-world data with limited sampling, despite being derived from simplifying modeling assumptions.

**Table 1.** Selection coefficient estimated from the *medionigra* morph dataset, assuming a codominant model. The data between the years 1939 and 1995 were obtained from Cook and Jones (1996), and the data after 1996 were obtained from Jones (2000). The 95% confidence interval (CI) for the MPL-based estimator $\hat{s}$ was computed using the likelihood ratio, which asymptotically follows a chi-square distribution (see Materials and Methods for details). Due to the use of a different fitness model in Jewett et al. (2016), their estimate was scaled, rendering its confidence interval inapplicable for comparison. We assumed a zero-mutation probability for inference.

Population data for years	Estimator	Selection coefficient estimate (95% CI)	Model accounts for	
			Genetic drift	Limited sampling
1939–1995	MPL-based estimator $\hat{s}$	− 0.049 (−0.101, 0.005)	✓	
	Jewett et al. (2016)	− 0.050 (n.a.)	✓	✓
1939–1999	MPL-based estimator $\hat{s}$	− 0.058 (−0.108, −0.004)	✓	
	Mathieson and McVean (2013)	− 0.057 (−0.113, −0.003)	✓	✓

These results show that, despite modeling only genetic drift (and not limited sampling), the MPL-based estimator $\hat{s}$ can efficiently provide selection coefficient estimates comparable to those obtained from an estimator that accounts for both sampling and drift, particularly when sufficiently long trajectories are available. While our analysis has focused on the MPL-based estimator, the observation that MPL- and HMM-based estimators exhibit comparable performance for sufficiently long trajectories (Fig. 7) suggests that the performance insights derived here may extend to a broader class of estimators.

## Discussion

The MPL framework, introduced in our previous work (Sohail et al. 2021), provides closed-form selection coefficient estimators for genetic time-series data. This expression, presented in (3), bears similarities to estimators derived in earlier studies (Watterson 1979, 1982; Mathieson and McVean 2013), even though these were developed using different statistical methodologies. The MPL-based approach additionally incorporates the effect of mutation (i.e. μ>0) and accommodates scenarios where temporal samples are collected across multiple generations ($\Delta t_{k}>1$).

The MPL framework is analytically tractable and, as we show in this study, amenable to statistical analysis. Our analysis reveals that the MPL-based estimator is effectively unbiased under both limited sampling and genetic drift effects, with its variance decomposing into sampling-only and drift-only components that can be studied separately. Both variance components decrease with the integrated variance *V* along the mutant allele frequency trajectory, following rates of $O(n_{s}^{-1}V^{-2})$ and $O(N^{-1}V^{-1})$, respectively. This integrated variance emerges as a pivotal quantity that increases with trajectory length *T*, provided that the locus remains polymorphic over the additional generations used for inference. Our findings therefore highlight the importance of observing mutant allele frequencies over a sufficiently long time period, during which the frequency remains away from the boundaries. In scenarios where selection is weak, sample size is limited, or population size is small, such an extension of the observation period for mutant allele frequencies can effectively reduce estimator uncertainties and enhance the accuracy of inferences.

For the MPL-based estimator, our statistical analysis reveals that as the trajectory length increases, genetic drift becomes the dominant factor in determining performance, surpassing the effect of limited sampling. One likely reason for this is that genetic drift effect is cumulative over a trajectory, whereas fluctuations due to limited sampling effect are independent at different time points. Our results are consistent with observations presented in Lynch and Ho (2020), where performance measures that do not account for the cumulative effect of genetic drift were found to underestimate actual performance for longer trajectory lengths.

Although limited sampling effect is not explicitly modeled in the derivation of the MPL-based approach, the estimator still demonstrates robustness to this effect. Indeed, when comparing the MPL-based estimator with approaches that do account for limited sampling in their derivations, such as HMM-based methods, we find comparable performance across a wide range of conditions in both simulated and real datasets. These findings may have broader implications for time-series selection inference: while most contemporary methods emphasize incorporating limited sampling effect in their derivations, our results suggest this additional modeling complexity—which often leads to analytically intractable and difficult to interpret estimators—may be unnecessary in practice, particularly when sufficient temporal data is available.

Our main results have focused on characterizing estimation performance. However, these results may also be applied in a detection context, such as defining the minimum selection strength that can be detected for a given set of evolutionary parameters or determining the evolutionary conditions that enable reliable detection of selection at a given strength. Under a Gaussian approximation for $\hat{s}$, the minimum selection coefficient $s_{\min}$ that is detectable at the 5% significance level is obtained by solving $\text{Var}[\hat{s}]=(s_{\min}/2{)}^{2}$ (Lynch and Ho 2020). The left-hand side can be estimated using the analytical expression (11), which was validated as an accurate approximation of the empirical estimator variance based on simulations (Supplementary Fig. S12). Our simulations further highlighted the benefits of operating over longer temporal trajectories, leading to significantly enhanced detection power. For example, for a population size N=1,000, increasing the trajectory length *T* from 50 to 150 generations more than halved the minimum detectable selection coefficient, reducing it from above 4% to below 2% (Supplementary Fig. S12).

The MPL-based estimator $\hat{s}$ analyzed in this study is derived under the WF model, which assumes a panmictic hermaphroditic population with a constant population size. Despite the simplicity and foundational nature of this evolutionary model, the relative contributions and interactions of sampling noise and genetic drift on selection coefficient estimators are not well understood. Our analysis provides a detailed examination of these effects; however, the WF model omits several factors that can substantially influence evolutionary dynamics in natural populations. For instance, population structure can affect allele frequency dynamics, and when detailed information about such structures is available, it could be integrated into the evolutionary model for selection inference (Balding 2006; Mathieson and McVean 2013). Additionally, selection coefficients can vary over time in biological systems, suggesting that future work could focus on analyzing models associated with time-varying selection. It is also important to note that the actual population size in natural populations is subject to temporal variation. While our study did not investigate the performance of the MPL-based estimator under time-varying population sizes, previous research has demonstrated that selection inference is surprisingly robust to such variations (Mathieson and McVean 2013; Foll et al. 2015; Jewett et al. 2016; Lee et al. 2025). Notably, Lee et al. (2025) reveal that even when the true population size fluctuates over time, inferring selection coefficients is more accurate when assuming a constant effective population size rather than incorporating the estimated time-varying population sizes. We emphasize that MPL is a general framework that can accommodate WF-like models with complexities such as multiple loci evolving under linkage effects (Sohail et al. 2021; Li and Barton 2023; Gao and Barton 2025), epistasis (Sohail et al. 2022), and time-varying population size (Lee et al. 2025). Furthermore, the MPL framework can integrate evolutionary dynamics beyond the WF model. For example, our recent work (Lee et al. 2025) employed a Galton–Watson-like branching process within the MPL framework to infer the transmission advantage of mutations in epidemiological contexts. In principle, the MPL framework is also flexible to allow for evolutionary models of dioecious populations. While our focus here has been on establishing performance analysis of the fundamental single-locus WF model, it paves the way for further research to characterize the performance of estimators derived from more complex models.

A key advantage of the MPL framework is that it yields an analytically tractable closed-form selection coefficient estimator. This estimator is derived by approximating the transition probabilities of the WF process with the transition probability density of a diffusion process and constructing the path likelihood of mutant allele frequencies, resulting in a Gaussian form. We note that the diffusion approximation is accurate under the standard diffusion regularity conditions (large *N*, constant *Ns* and Nμ, small Δt). Interestingly, previous studies have shown that moderate to large deviations from these regularity assumptions are well tolerated by the MPL-based selection coefficient estimators, resulting in only limited loss of accuracy (Sohail et al. 2021, 2022). However, substantial departures from the regularity conditions can render the Gaussian path likelihood an inaccurate approximation of the WF path likelihood, in which case moment-based approaches may provide more reliable estimates (Tataru et al. 2017; Paris et al. 2019).

We have analyzed the asymptotic performance of the MPL-based estimator in terms of the integrated variance *V*, a monotonically increasing quantity that grows as the trajectory length *T* (i.e. the number of generations used for inference) increases. When selection is strong, the frequency of the mutant allele increases rapidly, resulting in a corresponding rapid growth in *V*, and vice versa. Strictly speaking, the asymptotic analysis (in terms of *V*) assumes that *V* keeps growing with the trajectory length *T*; specifically, as long as increasing *T* leads to a growing value of *V*, the estimator performance improves. However, once the mutant allele frequency reaches fixation, *V* plateaus, and further increases in *T* yield no additional gains in estimator performance, particularly when the mutation probability from the mutant allele to the wild-type allele is low. This observation aligns with findings by Feder et al. (2014), noting that once a mutant allele frequency reaches fixation, any subsequent frequency observations become uninformative for inferring selection. Moreover, we acknowledge the limitation of assuming a symmetric mutation rate in our analysis, which may not always apply. Nonetheless, asymptotic analysis remains useful, providing tractable characterizations of estimator behavior, establishing fundamental performance benchmarks, and offering insights into estimator performance even when *V* is finite. Additionally, from the definition of *V* in (9), we note that points along the mutant allele frequency trajectory that are farther from the boundaries contribute more significantly to *V* than those that are close to the boundaries (0 or 1). Consequently, *V* is smaller for rapidly growing trajectories (large selection coefficients) than for slowly growing ones (small selection coefficients), as the latter set of trajectories includes more points that are away from the boundaries (Supplementary Fig. S3B).

Although the MPL framework was originally introduced in a Bayesian context (Sohail et al. 2021, 2022), here we have analyzed the MPL-based selection coefficient estimator under a maximum likelihood formulation for a more tractable performance analysis. While the Bayesian approach offers advantages such as incorporating prior information and quantifying uncertainty, analyzing the estimator mean and deriving the CRLB (required for analyzing the estimator variance) are considerably more challenging compared to those in maximum likelihood approaches. Nonetheless, our analysis lays the groundwork for future studies to examine the performance of the Bayesian selection coefficient estimator in greater detail.

Our analysis is restricted to single-locus models, which can serve as an approximation for practical evolutionary processes, as it assumes that loci evolve independently. This assumption is commonly applied in studies of time-series based selection estimation (Bollback and Huelsenbeck 2007; Mathieson and McVean 2013; Lacerda and Seoighe 2014; Jewett et al. 2016; Iranmehr et al. 2017; Taus et al. 2017). In reality, evolving populations can exhibit more complex dynamics with interactions occurring among multiple loci. MPL-based estimators have been derived for multi-locus systems and have been shown to account for confounding effects including genetic linkage (Sohail et al. 2021) and epistasis (Sohail et al. 2022). These multilocus MPL-based estimators have also demonstrated robust performance across simulated and real datasets (Sohail et al. 2021, 2022; Li and Barton 2023; Lee et al. 2025); however, a comprehensive statistical analysis of their performance has not yet been established. Extending the analysis presented in this work to multilocus models poses additional challenges, as it requires characterizing moments of higher-order mutant allele frequencies and the inverse of an integrated covariance matrix. Future research will aim to address these technical challenges and characterize the statistical properties of these multilocus estimators in greater depth. We anticipate that key insights that we have obtained in our analysis of single-locus models will carry over to more complex multilocus settings.

## Materials and Methods

### Selection Coefficient Estimator

The closed-form selection coefficient estimator we consider is derived under the MPL framework (Sohail et al. 2021, 2022). It is based on a maximum likelihood criterion and applied to a stochastic evolutionary model. We also provide correction for the bias associated with limited sampling effect which is not explicitly modeled in the MPL derivation.

#### Maximum Likelihood Selection Coefficient Estimator

We model the evolution of a population of *N* individuals as a Wright–Fisher (WF) process with selection and mutation, and consider a bi-allelic model where the wild-type and mutant alleles are represented by 0 and 1, respectively. The Wrightian fitness of the mutant allele is given by (1+s), where *s* is the selection coefficient. We denote the population mutant allele frequency at generation *t* by x(t), where x(t)=n(t)/N and n(t) is the number of individuals in the population with the mutant allele. Under the WF dynamics, the probability that the population mutant allele frequency is x(t+1) at generation (t+1), given population mutant allele frequency x(t) at generation *t*, is expressed as


(14) $$P(x(t+1)\;|\;x(t))=\left(\begin{array}{c} N\\ n(t+1) \end{array}\right)p{\left(t\right)}^{n(t+1)}(1-p(t){)}^{N-n(t+1)}$$

with


(15) $$p(t)=\frac{\left(1+s)x(t)+\mu [(1-x(t))-(1+s)x(t)\right]}{1+sx(t)},$$

where *μ* is the mutation probability per generation.

The probability that the population mutant allele frequency follows a particular trajectory $\left(x(t_{1}),x(t_{2}),\ldots ,x(t_{K})\right)$ given $x(t_{0})$, where $t_{k}$ are nonconsecutive generations with k∈{0,1,…,K}, is given as


(16) $$P\left({\left(x(t_{k})\right)}_{k=1}^{K}\;|\;x(t_{0})\right)=\prod_{k=0}^{K-1}P\left(x(t_{k+1})\;|\;x(t_{k})\right).$$

The MPL framework efficiently quantifies the probability in (16) by a path integral. This is achieved by making use of the diffusion approximation of the WF process (Ewens 2004; Durrett 2008; Bollback et al. 2008; Malaspinas et al. 2012; Steinrücken et al. 2014; Ferrer-Admetlla et al. 2016; Schraiber et al. 2016; Sohail et al. 2021; Sant et al. 2022; He et al. 2023; Jenkins 2024, unpublished data; Sant et al. 2025, unpublished data). Under this approximation, both the selection coefficient *s* and mutation probability *μ* are assumed of order $O(N^{-1})$, and the transition probabilities on the right-hand side in (16) can be expressed as the transition probability density of the diffusion process (Risken 1989) scaled by a constant. Next, the MPL framework discretizes the transition probability density for small time steps, which results in a Gaussian form of the transition density (full details of this approach are given in Sohail et al. 2021). The probability that the population mutant allele frequency at generation $t_{k+1}$ is $x(t_{k+1})$ given the frequency at the preceding time point $t_{k}$ is expressed as


(17) $$P\left(x(t_{k+1})\;|\;x(t_{k}),N,\mu ,s\right)\approx dx(t_{k+1})\;\phi \left(x(t_{k+1})\;|\;x(t_{k})\right),$$

where $dx(t_{k+1})$ represents the small frequency difference that accounts for the quantization of continuous frequency space. The term $\phi (x(t_{k+1})\;|\;x(t_{k}))$ in (17) is given as


(18) $$\phi \left(x(t_{k+1})\;|\;x(t_{k})\right)\approx \sqrt{\frac{N}{2\pi \Delta t_{k}v(t_{k})}}\exp\;\left[-\frac{N}{2}\Theta (x(t_{k+1}),x(t_{k}))\right],$$

with $\Delta t_{k}=t_{k+1}-t_{k}$ denoting the time sampling step between generations $t_{k}$ and $t_{k+1}$. The term $\Theta (x(t_{k+1}),x(t_{k}))$ in (18) is written as


(19) $$\Theta (x(t_{k+1}),x(t_{k}))=\frac{{\left[x(t_{k+1})-x(t_{k})-\Delta t_{k}(sv(t_{k})+\mu (1-2x(t_{k})))\right]}^{2}}{\Delta t_{k}v(t_{k})},$$

with


(20) $$v(t_{k})=x(t_{k})\left(1-x(t_{k})\right)$$

defined as the population mutant allele variance at generation $t_{k}$.

We use the maximum likelihood criterion to obtain an estimate of the selection coefficient $\hat{s}$, given the population mutant allele frequency trajectory $\left(x(t_{0}),x(t_{1}),\ldots ,x(t_{K})\right)$, the population size *N*, and the mutation probability per generation *μ*. The selection coefficient estimator is thus obtained by solving


(21) $${\hat{s}}_{\mathrm{MPL}}=\underset{s}{\arg\;\max}\;L(s\;|\;{\left(x(t_{k})\right)}_{k=0}^{K},N,\mu ),$$

where the likelihood function $L(s\;|\;(x(t_{k}){)}_{k=0}^{K},N,\mu )$ is given by


(22) $$\begin{array}{rl} L(s\;|\;{\left(x(t_{k})\right)}_{k=0}^{K},N,\mu ) & =P\left((x(t_{k}){)}_{k=1}^{K}\;|\;x(t_{0}),N,\mu ,s\right)\\ & =\prod_{k=0}^{K-1}P\left(x(t_{k+1})\;|\;x(t_{k}),N,\mu ,s\right). \end{array}$$

Solving (21) yields the following closed-form solution


(23) $${\hat{s}}_{\mathrm{MPL}}=\frac{x(t_{K})-x(t_{0})-\mu \sum_{k=0}^{K-1}\Delta t_{k}\left(1-2x(t_{k})\right)}{\sum_{k=0}^{K-1}\Delta t_{k}v(t_{k})}.$$

#### Selection Coefficient Estimator Incorporating Observed Mutant Allele Frequencies

The selection coefficient estimator ${\hat{s}}_{\mathrm{MPL}}$ (23) relies on knowledge of the population mutant allele frequency trajectory. In practice, these population frequencies are estimated by sampling the population. Here, we consider each observation of mutant allele frequency as a random variable, and substitute the observed mutant allele frequencies in place of population mutant allele frequencies in (23).

Given the population mutant allele frequency $x(t_{k})$ and the sample size $n_{s}(t_{k})$, we assume that the distribution of mutant allele count $c(t_{k})$ is binomial, as is commonly assumed in the population genetics literature (Mathieson and McVean 2013; Jewett et al. 2016; Iranmehr et al. 2017; He et al. 2020). The probability of observing mutant allele frequency $\hat{x}(t_{k})$ is expressed as


(24) $$\begin{array}{r} P(\hat{x}\left(t_{k})\;|\;x(t_{k}),n_{s}(t_{k})\right)=\left(\begin{array}{c} n_{s}(t_{k})\\ c(t_{k}) \end{array}\right)\;x(t_{k}{)}^{c(t_{k})}\;(1-x(t_{k}){)}^{n_{s}(t_{k})-c(t_{k})}, \end{array}$$

where $\hat{x}(t_{k})=c(t_{k})/n_{s}(t_{k})$ with mean $x(t_{k})$ and variance $v(t_{k})/n_{s}(t_{k})$ (see Supplementary Information for details). Substituting these observed frequencies in the estimator ${\hat{s}}_{\mathrm{MPL}}$ (23) gives the selection coefficient estimator under limited sampling effect, $\hat{s}$, as


(25) $$\hat{s}=\frac{\hat{x}(t_{K})-\hat{x}(t_{0})-\mu \sum_{k=0}^{K-1}\Delta t_{k}\left(1-2\hat{x}(t_{k})\right)}{\sum_{k=0}^{K-1}\left(\frac{n_{s}(t_{k})}{n_{s}(t_{k})-1}\right)\Delta t_{k}\hat{v}(t_{k})}=\frac{\hat{D}}{\hat{V}},$$

where $\hat{v}(t_{k})=\hat{x}(t_{k})(1-\hat{x}(t_{k}))$ is the observed (sample) mutant allele variance. The correction factor $n_{s}(t_{k})/\left(n_{s}(t_{k})-1\right)$ accounts for the fact that $\hat{v}(t_{k})$ is a biased estimate of the population mutant allele variance $v(t_{k})$ (see Supplementary Information for details).

The estimator $\hat{s}$ is a ratio of two correlated random variables, $\hat{D}$ and $\hat{V}$, where the correlation arises because the frequencies of observed mutant alleles are shared between these two random variables. Here, $\hat{D}$ represents the difference between the net change in the observed mutant allele frequency trajectory, $\hat{x}(t_{K})-\hat{x}(t_{0})$, adjusted for changes due to mutational effect, and $\hat{V}$ represents the bias-corrected observed integrated variance along the mutant allele frequency trajectory.

### Analysis under Limited Sampling but no Genetic Drift (Deterministic Evolutionary Model)

For our analysis of the selection coefficient estimator $\hat{s}$ (25) in the absence of genetic drift, we consider a discrete-time single-locus deterministic evolutionary model with selection and mutation, where sampling noise is the only source of randomness. This model is equivalent to the WF model in the limit of infinite population size *N*. We first demonstrate that with the deterministic evolutionary model, if limited sampling is not explicitly modeled (as above), we arrive at the same estimator form as (25).

Under the deterministic evolutionary model, given the frequency at generation *t*, the mutation probability *μ*, and the selection coefficient *s*, the deterministic population mutant allele frequency at generation (t+1) can be computed as


(26) $$x(t+1)=\frac{\mu (1-x(t))+(1+s)(1-\mu )x(t)}{1+sx(t)}.$$

We assume that both the selection coefficient *s* and the mutation probability *μ* are small, such that higher order terms involving *s* and *μ* can be neglected. By performing a Taylor expansion of the right-hand side of the preceding equation with respect to *s*, and subsequently retaining only the leading-order terms, we obtain the simplified expression


(27) x(t+1)=x(t)+sv(t)+μ(1−2x(t)),

where v(t)=x(t)(1−x(t)) is the population mutant allele variance at generation *t*. Iterating the above expression $\Delta t_{k}$ times, the population mutant allele frequency at generation $t_{k+1}=t_{k}+\Delta t_{k}$ given the mutant allele frequency at generation $t_{k}$ can be written as


(28) $$x(t+\Delta t_{k})=x(t_{k})+\Delta t_{k}\left[sv(t_{k})+\mu (1-2x(t_{k}))\right].$$

Integrating both sides of (28) over the population mutant allele frequency trajectory $\left(x(t_{0}),x(t_{1}),\ldots ,x(t_{K})\right)$ and simplifying gives


(29) $$x(t_{K})-x(t_{0})=s\sum_{k=0}^{K-1}\Delta t_{k}v(t_{k})+\mu \sum_{k=0}^{K-1}\Delta t_{k}(1-2x(t_{k})).$$

Therefore, the selection coefficient *s* of a population evolving under this deterministic evolutionary model can be obtained by rearranging the above expression, i.e.


(30) $$s=\frac{x(t_{K})-x(t_{0})-\mu \sum_{k=0}^{K-1}\Delta t_{k}(1-2x(t_{k}))}{\sum_{k=0}^{K-1}\Delta t_{k}v(t_{k})}.$$

As anticipated, the right-hand side in (30) has the same form as the maximum likelihood estimator ${\hat{s}}_{\mathrm{MPL}}$ obtained in (23) under the stochastic evolutionary model. Consequently, after applying bias correction to $\hat{V}$, the estimator under limited sampling effect shares the same expression as $\hat{s}$ (25) .

#### Estimator Mean and Variance

Considering the effect of limited sampling, for a deterministic population mutant allele trajectory, the observed mutant allele frequency trajectory comprises a set of independent random variables $\left(\hat{x}(t_{0}),\hat{x}(t_{1}),\ldots ,\hat{x}(t_{K})\right)$. With this trajectory, the selection coefficient estimator $\hat{s}$ under the deterministic evolutionary model has the same form as in (25) and is a ratio of two random variables. We approximate these quantities using a multivariate Taylor series expansion with respect to the numerator $\hat{D}$ and the denominator $\hat{V}$ about their expectations $\text{E}[\hat{D}]$ and $\text{E}[\hat{V}]$ (see Casella and Berger 2002). To simplify the analysis and lighten notation, we will assume a constant sample size $n_{s}$ and a time sampling step Δt. Considering terms up to the first-order of this expansion around $\text{E}[\hat{D}]$ and $\text{E}[\hat{V}]$ gives the mean of selection coefficient estimator as


(31) $$\begin{array}{rl} \text{E}[\hat{s}] & =\text{E}\left[\frac{\hat{D}}{\hat{V}}\right]\\ & \approx \frac{\text{E}[\hat{D}]}{\text{E}[\hat{V}]}\\ & =\frac{x(t_{K})-x(t_{0})-\mu \Delta t\sum_{k=0}^{K-1}\left(1-2x(t_{k})\right)}{\Delta t\sum_{k=0}^{K-1}v(t_{k})}. \end{array}$$

The last line follows from the fact that $\hat{D}$ and $\hat{V}$ are linear combinations of the observed mutant allele frequencies and the bias-corrected observed mutant allele variances respectively (see Supplementary Information for details). Note that the right-hand side in (31) has the same form as the estimator ${\hat{s}}_{\mathrm{MPL}}$ obtained in (23) and is effectively the true selection coefficient *s* as indicated by (30), i.e.


(32) $$\text{E}[\hat{s}]={\hat{s}}_{\mathrm{MPL}}=s.$$

Denoting the integrated variance of the population mutant allele frequency trajectory by *V*, i.e.


(33) $$V\;:=E[\hat{V}]=\Delta t\sum_{k=0}^{K-1}v(t_{k}),$$

and using the first-order multivariate Taylor series expansion along with (31), the variance of the selection coefficient estimator due to limited sampling effect, Var[$\hat{s}$], can be written as (see, Example 5.5.27 in Casella and Berger 2002)


(34) $$\begin{array}{rl} \text{Var}[\hat{s}] & =\frac{\text{Var}[\hat{D}]}{{\text{E}}^{2}[\hat{V}]}-2\frac{\text{E}[\hat{D}]}{\text{E}[\hat{V}]}\frac{\text{Cov}[\hat{D},\hat{V}]}{{\text{E}}^{2}[\hat{V}]}+{\left(\frac{\text{E}[\hat{D}]}{\text{E}[\hat{V}]}\right)}^{2}\frac{\text{Var}[\hat{V}]}{{\text{E}}^{2}[\hat{V}]}\\ & =\frac{\text{Var}[\hat{D}]}{V^{2}}-2{\hat{s}}_{\mathrm{MPL}}\frac{\text{Cov}[\hat{D},\hat{V}]}{V^{2}}+{\hat{s}}_{\mathrm{MPL}}^{2}\frac{\text{Var}[\hat{V}]}{V^{2}}. \end{array}$$

Here, $\text{Var}[\hat{D}]$, $\text{Cov}[\hat{D},\hat{V}]$, and $\text{Var}[\hat{V}]$ are given by (see Supplementary Information for details)


(35) $$\text{Var}[\hat{D}]=\frac{1}{n_{s}}\left[v(t_{K})+v(t_{0})-4\mu \Delta tv(t_{0})+4\mu^{2}\Delta tV\right],$$

while


(36) $$\text{Cov}[\hat{D},\hat{V}]=-\frac{\Delta t}{n_{s}}\times \left[v(t_{0})\left(1-2x(t_{0})\right)-2\mu \Delta t\sum_{k=0}^{K-1}v(t_{k})\left(1-2x(t_{k})\right)\right],$$

and


(37) $$\text{Var}[\hat{V}]=\frac{\Delta t}{n_{s}}\left[V-\left(4-\frac{2}{n_{s}-1}\right)\left(\Delta t\sum_{k=0}^{K-1}v^{2}(t_{k})\right)\right].$$

#### Order Analysis of Estimator Variance

Insights into the robustness of the estimator $\hat{s}$ against limited sampling effect are provided by characterizing the scaling behavior of the estimator variance (34) with respect to sample size $n_{s}$ and the integrated variance *V*. Noting from (32) and recalling that both (23) and (30) were derived under the assumption that the true selection coefficient *s* and the mutation probability *μ* were small, we assume that the selection coefficient estimate ${\hat{s}}_{\mathrm{MPL}}$ is also small. We additionally assume that the population mutant allele frequency is away from the boundaries at most time points along the trajectory. Under these assumptions, the first term on the right-hand side of (34) can be written as


(38) $$\begin{array}{rl} \frac{\text{Var}[\hat{D}]}{V^{2}} & =\frac{1}{n_{s}}\left[\frac{v(t_{K})+v(t_{0})}{V^{2}}-4v(t_{0})\Delta t\frac{\mu }{V^{2}}\right]\\ & =O(n_{s}^{-1}V^{-2}), \end{array}$$

where $\text{Var}[\hat{D}]$ is given by (35), and the temporal population mutant allele variances, $v(t_{0})$ and $v(t_{K})$, are bounded with order O(1). Using (36), we can write the second term on the right-hand side of (34) as


(39) $$\begin{array}{rl} -2{\hat{s}}_{\mathrm{MPL}}\frac{\text{Cov}[\hat{D},\hat{V}]}{V^{2}} & =\frac{\Delta t}{n_{s}V^{2}}\times 2{\hat{s}}_{\mathrm{MPL}}v(t_{0})(1-2x(t_{0}))\\ & =O(n_{s}^{-1}V^{-2}). \end{array}$$

The third term in (34) is dropped as we assume ${\hat{s}}_{\mathrm{MPL}}$ is small such that its higher-order terms can be neglected. Taking (38) and (39) together with (34), we can express the order of variance of $\hat{s}$ due to limited sampling as


(40) $$\text{Var}[\hat{s}]=O(n_{s}^{-1}V^{-2}).$$

#### Asymptotic Distribution

To characterize the distribution of $\hat{s}$ (25) under limited sampling effect, we assume that the sample size $n_{s}$ is sufficiently large and the frequency trajectory is away from the boundaries. Under these assumptions, the observed mutant allele frequency $\hat{x}(t_{k})$ can be approximated as a Gaussian random variable. The numerator $\hat{D}$ is then also Gaussian, since it is a linear combination of Gaussian variables. The denominator $\hat{V}$ is the sum of *K* independently observed bias-corrected mutant allele variances at generations $\left(t_{0},t_{1},\ldots ,t_{K-1}\right)$, where all observed mutant allele variances are bounded on (0,0.25] but not necessarily drawn from identical distributions. We consider a scenario where the integrated variance *V* is obtained from a sufficiently long trajectory (i.e. large *T*), with a large number of observations *K*. As *V* approaches infinity, the denominator $\hat{V}$ converges in distribution to a Gaussian random variable according to the Lyapunov central limit theorem (see Supplementary Information). The ratio of Gaussian random variables can be approximated as Gaussian when the coefficient of variation of the denominator approaches zero, as shown by the analytical results from Hinkley (1969). Using this approximation, the estimator $\hat{s}$ (25) can be shown to be asymptotically Gaussian as the integrated variance *V* approaches infinity (see Supplementary Information for details).

### Analysis under Limited Sampling and Genetic Drift

#### Estimator Mean

Considering the joint effect of limited sampling and genetic drift, according to the law of total expectation, the mean of $\hat{s}$ (25) can be written as


(41) $$\text{E}[\hat{s}]={\text{E}}_{d}\left[{\text{E}}_{s}\left[\hat{s}\;|\;(x(t_{k}){)}_{k=0}^{K}\right]\right],$$

where ${\text{E}}_{s}[\hat{s}\;|\;(x(t_{k}){)}_{k=0}^{K}]$ denotes the estimator mean with respect to fluctuations due to limited sampling, given a realization of a WF population mutant allele frequency trajectory $(x(t_{k}){)}_{k=0}^{K}$, and ${\text{E}}_{d}$ denotes the mean with respect to stochastic WF trajectories.

Given the WF trajectory $(x(t_{k}){)}_{k=0}^{K}$, the observed mutant allele frequencies can be treated as independent random variables. This allows us to derive an analytical expression for the conditional mean ${\text{E}}_{s}[\hat{s}\;|\;(x(t_{k}){)}_{k=0}^{K}]$, considering a first-order multivariate Taylor series expansion, as


(42) $$\begin{array}{rl} {\text{E}}_{s}\left[\hat{s}\;|\;(x(t_{k}){)}_{k=0}^{K}\right] & =\frac{x(t_{K})-x(t_{0})-\mu \Delta t\sum_{k=0}^{K-1}\left(1-2x(t_{k})\right)}{\Delta t\sum_{k=0}^{K-1}v(t_{k})}\\ & ={\hat{s}}_{\mathrm{MPL}}, \end{array}$$

where the last line follows the maximum likelihood estimator ${\hat{s}}_{\mathrm{MPL}}$ derived in (23). Therefore, the estimator mean E[$\hat{s}$] in (41) can be written as


(43) $$\text{E}[\hat{s}]={\text{E}}_{d}\left[{\hat{s}}_{\mathrm{MPL}}\right],$$

which indicates that E[$\hat{s}$] under the current stochastic evolutionary model is effectively the mean of estimator ${\hat{s}}_{\mathrm{MPL}}$ under only the genetic drift effect. Our analysis further shows that the mean of estimator ${\hat{s}}_{\mathrm{MPL}}$ can be computed as


(44) $$\begin{array}{rl} {\text{E}}_{d}\left[{\hat{s}}_{\mathrm{MPL}}\right] & ={\text{E}}_{d}\left[\frac{x(t_{K})-x(t_{0})-\mu \Delta t\sum_{k=0}^{K-1}\left(1-2x(t_{k})\right)}{\Delta t\sum_{k=0}^{K-1}v(t_{k})}\right]\\ & \approx \frac{{\text{E}}_{d}\left[x(t_{K})-x(t_{0})-\mu \Delta t\sum_{k=0}^{K-1}\left(1-2x(t_{k})\right)\right]}{{\text{E}}_{d}\left[\Delta t\sum_{k=0}^{K-1}v(t_{k})\right]}\\ & =s, \end{array}$$

where the second step uses the first-order multivariate Taylor series expansion with respect to the numerator and the denominator about their mean, and the last step is obtained by evaluating the mean of the numerator and the denominator (see Supplementary Information for details). From (43) and (44), we obtain


(45) $$\text{E}[\hat{s}]={\text{E}}_{d}[{\hat{s}}_{\mathrm{MPL}}]=s,$$

which implies that the estimator $\hat{s}$ under the joint effect of limited sampling and genetic drift is effectively unbiased.

#### Estimator Variance

Analyzing the joint effect of limited sampling and genetic drift on the estimator variance is challenging. To simplify the analysis, we make use of the empirical observation (11) that the estimator variance Var[$\hat{s}$] is approximately the sum of sampling-only variance $\sigma_{s}^{2}[\hat{s}]$ and drift-only variance $\sigma_{d}^{2}[\hat{s}]$ (e.g. Fig. 5b). This allows us to study their effects on estimator variance separately. Since the mean trajectory under the WF model closely tracks the population mutant allele frequency trajectory under the deterministic evolutionary model (Supplementary Fig. S8), the sampling-only variance follows the same asymptotic property as the estimator variance indicated in (40).

To characterize the contribution of genetic drift to the estimator variance, we derive the Cramér-Rao lower bound (CRLB) for the drift-only variance. The CRLB provides a theoretical minimum variance of an estimator (Kay 1993). For this analysis, we consider that the population mutant allele frequency is known so we can study the effect of genetic drift in isolation from limited sampling. In this case, the estimator $\hat{s}$ is equivalent to the estimator ${\hat{s}}_{\mathrm{MPL}}$ (23). Following similar analysis steps as those used in (44), the estimator $\hat{s}$ turns out to be unbiased under genetic drift effect. The CRLB, for an unbiased estimator, bounds the estimator variance by the reciprocal of the Fisher Information I(s), i.e.


(46) $$\sigma_{d}^{2}[\hat{s}]\geq I^{-1}(s).$$

The Fisher Information is defined as


(47) $$I(s)=-\text{E}\left[\frac{\partial^{2}}{\partial s^{2}}\ell (s)\right],$$

where $\ell (s)=\log\;P((x(t_{k}){)}_{k=1}^{K}\;|\;x(t_{0}),N,\mu ,s)$ denotes the log-likelihood of the population mutant allele frequency following the trajectory $(x(t_{k}){)}_{k=1}^{K}$ given initial population mutant allele frequency $x(t_{0})$, population size *N*, mutation probability *μ*, and selection coefficient *s*. In the MPL framework, the second derivative of ℓ(s) with respect to *s* can be written as (see Supplementary Information for details)


(48) $$\frac{\partial^{2}}{\partial s^{2}}\ell (s)=-N\Delta t\sum_{k=0}^{K-1}v(t_{k}),$$

and as such


(49) $$I(s)=N\;\text{E}\left[\Delta t\sum_{k=0}^{K-1}v(t_{k})\right].$$

Hence, the Fisher Information I(s) is equal to the product of population size and expected integrated variance, where the expectation is with respect to the stochastic WF population mutant allele frequency trajectories. Since the mean population mutant allele frequency trajectory under the WF model closely matches the frequency trajectory under the deterministic evolutionary model (Supplementary Fig. S8), we approximate $\text{E}[\Delta t\sum_{k=0}^{K-1}v(t_{k})]$ in (49) as the integrated variance *V* defined in (9). This gives the CRLB on the drift-only variance:


(50) $$\sigma_{d}^{2}[\hat{s}]\geq I^{-1}(s)=\frac{1}{NV}.$$

### Estimator Derived by Accounting for Sampling Effect

We obtain a maximum likelihood estimator ${\hat{s}}_{\mathrm{LS}}$ using a HMM. This estimator can be seen as an extension of the MPL-based approach to account for limited sampling in addition to genetic drift effect.

Given the initial mutant allele frequency $x(t_{0})$, the likelihood of obtaining an observed mutant allele frequency trajectory $\left(\hat{x}(t_{1}),\hat{x}(t_{2}),\ldots ,\hat{x}(t_{K})\right)$ is expressed as


(51) $$L(s)=\sum_{x(t_{1})\ldots x(t_{K})}\prod_{k=0}^{K-1}P(x(t_{k+1})\;|\;x(t_{k}),N,\mu ,s)\prod_{k=1}^{K}P(\hat{x}(t_{k})\;|\;x(t_{k}),n_{s}),$$

where $P(x(t_{k+1})\;|\;x(t_{k}),N,\mu ,s)$ is the transition probability function and $P(\hat{x}(t_{k})\;|\;x(t_{k}),n_{s})$ is the emission probability function. To reduce the computational burden associated with the large number of states in the frequency space, we follow the common practice of partitioning the mutant allele frequency space into a grid (Bollback et al. 2008; Malaspinas et al. 2012; Mathieson and McVean 2013; He et al. 2020). Here, we take a uniformly spaced grid $G=(g_{1},g_{2},\ldots ,g_{D})$ with D=100 and midpoints $\left(m_{1},m_{2},\ldots ,m_{D}\right)$, respectively, similar to Mathieson and McVean (2013). Given the population size *N*, mutation probability *μ*, and that the mutant allele frequencies at generations $t_{k}$ and $t_{k+1}$ fit into $g_{i}$ and $g_{j}$, respectively, based on the MPL framework, the transition probability is approximated as


(52) $$P\left(x(t_{k+1})\in g_{j}\;|\;x(t_{k})\in g_{i},N,\mu ,s\right)\approx \frac{1}{D}\;\phi \left(m_{j}\;|\;m_{i}\right),$$

where ϕ(⋅) is given in (18). The emission probabilities reflect incomplete sampling which, as in (24), are modeled as binomial:


$$P(\hat{x}(t_{k})\;|\;x(t_{k}),n_{s})=\left(\begin{array}{c} n_{s}\\ c(t_{k}) \end{array}\right)\;x^{c(t_{k})}(t_{k})\;(1-x(t_{k}){)}^{n_{s}-c(t_{k})},$$

where $c(t_{k})$ denotes the count of mutant alleles observed from $n_{s}$ samples at generation $t_{k}$.

Based on this HMM, we compute the logarithm of the likelihood L(s) in (51) using the forward algorithm implemented in the *hmmlearn* package (https://github.com/hmmlearn). The maximum likelihood estimate ${\hat{s}}_{\mathrm{LS}}$ is then obtained using the Nelder-Mead simplex algorithm (Nelder and Mead 1965) implemented in the *SciPy* package (Virtanen et al. 2020), with a tolerance of $10^{-4}$.

### Real-world Dataset

We used a classic real-world dataset to test whether a lack of explicitly accounting for limited sampling effect by the MPL framework can lead to inaccurate selection coefficient estimates. This dataset describes the evolution of the *medionigra* morph in a population of scarlet tiger moths (*Callimorpha dominula*). The *medionigra* phenotype is characterized by a reduction in the number and size of white/cream spots on the moth wings. The population of scarlet moths at Cothill Fen near Oxford was observed for the *medionigra* morph and recorded for 52 years between 1939 and 1999 (Cook and Jones 1996; Jones 2000), with one generation per year. This dataset has been analyzed in literature (Cook and Jones 1996; Mathieson and McVean 2013; Jewett et al. 2016) under the assumptions of a codominant model and a constant selection coefficient. The sample sizes range from 24 to 4,580, with 19 out of 52 observations having fewer than 500 samples. For our analysis, we defined the fitness of typical homozygote *AA* as 1, heterozygote *Aa* as (1+s), and rare homozygote *aa* as (1+2s). For consistency with published analysis that we compare with, we adopted a population size estimate of 2N=1,000.

### Estimator Confidence Interval

We construct a 95% confidence interval for the MPL-based estimator $\hat{s}$ (25) using the property that the likelihood ratio test statistic is asymptotically $\chi_{1}^{2}$ distributed (see, for example, Theorem 10.3.1 in Casella and Berger 2002 for more details). Specifically, the 95% confidence interval consists of all values of the selection coefficient $s_{0}$ that satisfy


(53) $$-2\left[\log\;L(s_{0})-\log\;L(\hat{s})\right]<3.84,$$

where L(⋅) is the likelihood function given in (22). The confidence interval is computed by substituting the population allele frequencies $x(t_{k})$ with observed frequencies $\hat{x}(t_{k})$. This confidence interval is taken as an approximation to the actual confidence interval, since the MPL-based likelihood function L(⋅) in (22) reflects uncertainties associated with genetic drift effect but does not explicitly account for those due to limited sampling.

## Supplementary Material

msaf301_Supplementary_Data

## Data Availability

Simulation data and Python implementations for reproducing the results presented in the article are freely available at https://github.com/qb-cheng/SL-MPL.
