# Supplementary material for: Selection Estimation from Genetic Time-Series Data: Effects of Limited Sampling and Genetic Drift
Source: Mol Biol Evol. 2025 Nov 28;42(12):msaf301. doi: 10.1093/molbev/msaf301 (PMC13223747; doi:10.1093/molbev/msaf301)
Supplement: msaf301_Supplementary_Data [file msaf301_supplementary_data.pdf]

Supplementary Information

Selection estimation from genetic time-series data: Effects of limited sampling and genetic drift

Qingbei Cheng[1,†], Muhammad Saqib Sohail[2,†,*], and Matthew R. McKay[3,4,*]

[1]Department of Electronic and Computer Engineering, Hong Kong University of Science and Technology, Hong Kong SAR, China

[2]Department of Computer Science, Bahria University, Lahore, Pakistan

[3]Department of Electrical and Electronic Engineering, University of Melbourne, Melbourne, Victoria, Australia

[4]Department of Microbiology and Immunology, University of Melbourne, at The Peter Doherty Institute for Infection and Immunity, Melbourne, Victoria, Australia

[†]Authors with equal contributions

[*]Corresponding authors: matthew.mckay@unimelb.edu.au and saqibsohail.bulc@bahria.edu.pk

Supplementary Figures

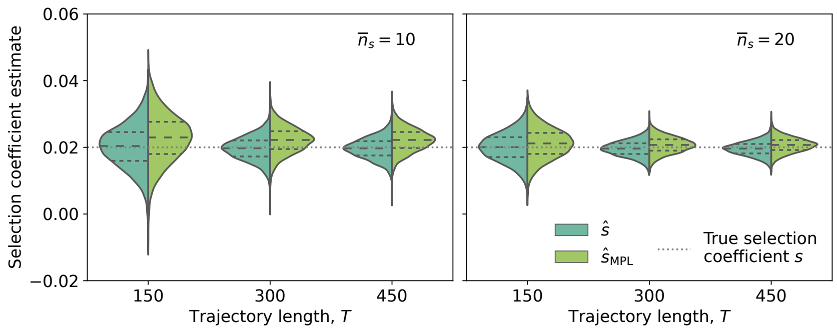

Figure S1 The bias correction term $\left(\frac{n_s(t_k)}{n_s(t_k)-1}\right)$ on the observed mutant allele variance $\hat{v}(t_k)$ in the estimator $\hat{s}$ improves the estimator performance under limited sampling conditions. The *left* side of violin plots shows the empirical distribution of the estimates $\hat{s}$ (5) obtained from 10^4 observations of a population frequency trajectory simulated under the deterministic evolutionary model, while the *right* side shows that of the estimates $\hat{s}_{\text{MPL}}$ without the bias-correction term as given in (3). The empirical distributions were shown for trajectory length $T = (150, 300, 450)$. The sample size at each time point is a Poisson random variable with mean value of $\overline{n}_s = (10, 20)$, respectively. Simulation parameters were selection coefficient $s = 0.02$, initial mutant allele frequency $x(0) = 0.1$, mutation probability $\mu = 10^{-3}$, with a constant time sampling step $\Delta t_k = 10$ for $k = (0, 1, \ldots, K-1)$.

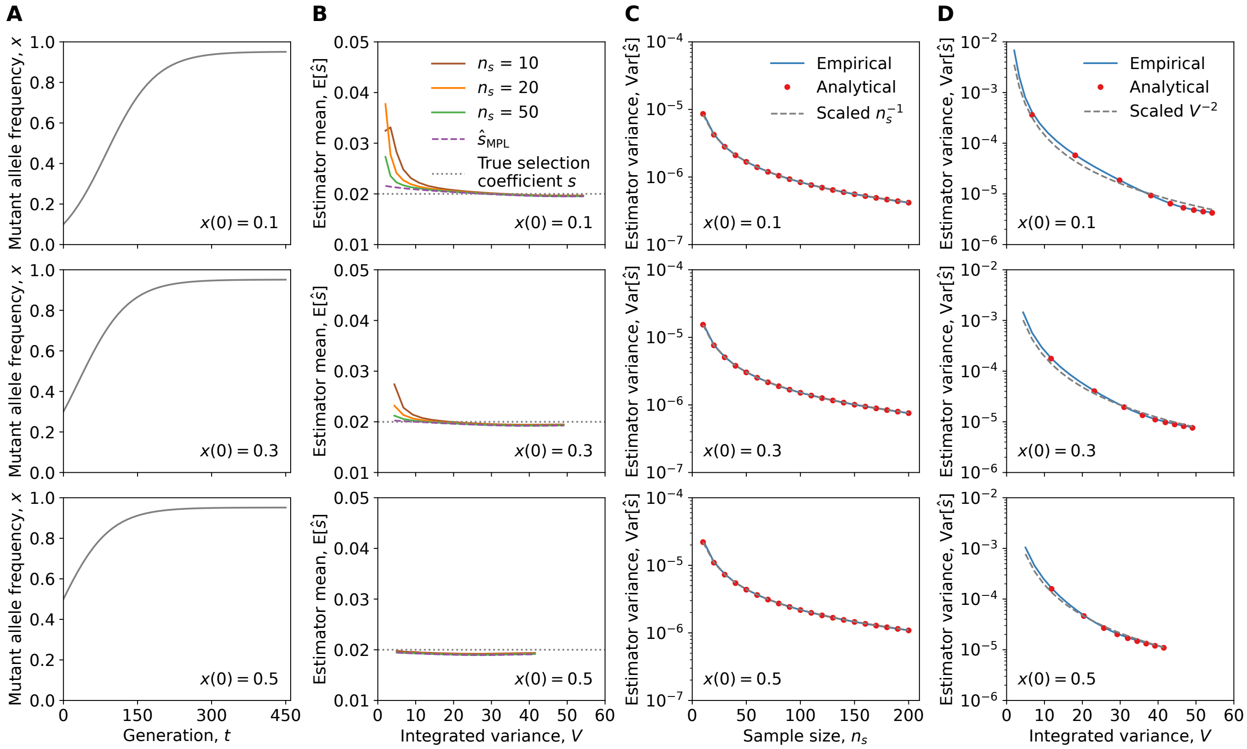

Figure S2 Our analytical expressions for the estimator mean (6) and variance (8) accurately reflect the empirical results under the deterministic evolutionary model with various initial mutant allele frequencies. (A) shows the simulated population mutant allele frequency trajectories. (B) shows the empirical estimator mean E[$\hat{s}$] with sample sizes $n_s = (10, 20, 50)$, the estimate $\hat{s}_{\text{MPL}}$ (3) obtained from the population mutant allele frequency trajectory without limited sampling effect, along with the true selection coefficient s. (C) shows that the estimator variance Var[$\hat{s}$] is of order $\mathcal{O}(n_s^{-1})$, where we fixed $T = 450$ (thus fixed the integrated variance V) and plotted the empirical estimator variance with respect to sample sizes n_s between 10 and 200. Values of the analytical estimator variance were obtained following (34) presented in Materials and Methods. (D) shows that the estimator variance Var[$\hat{s}$] is of order $\mathcal{O}(V^{-2})$, where we fixed $n_s = 50$ and plotted the empirical estimator variance with respect to integrated variance values V by varying T. Values of the analytical estimator variance were plotted when $T = (50, 100, \ldots, 450)$. Simulation parameters were selection coefficient $s = 0.02$, initial frequency $x(0) = (0.1, 0.3, 0.5)$ for each row respectively, mutation probability $\mu = 10^{-3}$, with time sampling step $\Delta t = 10$. Empirical values of estimator mean and variance were obtained from 10^6 observations of the population mutant allele frequency trajectory.

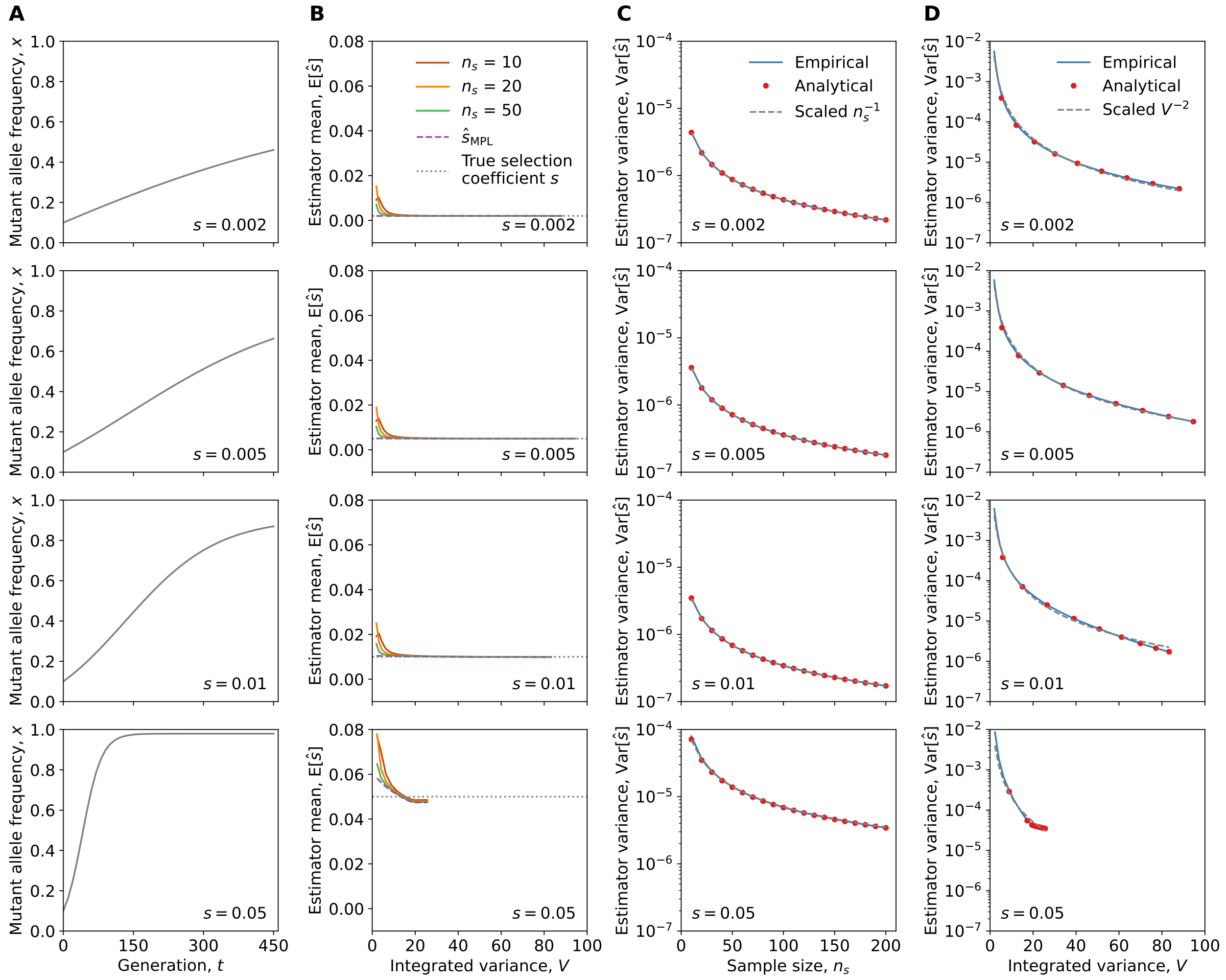

Figure S3 Our analytical expressions for the estimator mean (6) and variance (8) accurately reflect the empirical results under the deterministic evolutionary model with various selection coefficients. (A) shows the simulated population mutant allele frequency trajectories. (B) shows the empirical estimator mean $E[\hat{s}]$ with sample sizes $n_s = (10, 20, 50)$, the estimate $\hat{s}_{MPL}$ (3) obtained from the population mutant allele frequency trajectory without limited sampling effect, along with the true selection coefficient s. (C) shows that the estimator variance $Var[\hat{s}]$ is of order $\mathcal{O}(n_s^{-1})$, where we fixed $T = 450$ (thus fixed the integrated variance V) and plotted the empirical estimator variance with respect to sample sizes n_s between 10 and 200. Values of the analytical estimator variance were obtained following (34) presented in Materials and Methods. (D) shows that the estimator variance $Var[\hat{s}]$ is of order $\mathcal{O}(V^{-2})$, where we fixed $n_s = 50$ and plotted the empirical estimator variance with respect to integrated variance values V by varying T. Values of the analytical estimator variance were plotted when $T = (50, 100, \ldots, 450)$. Simulation parameters were selection coefficient $s = (0.002, 0.005, 0.01, 0.05)$ for each row respectively, initial frequency $x(0) = 0.1$, mutation probability $\mu = 10^{-3}$, with time sampling step $\Delta t = 10$. Empirical values of estimator mean and variance were obtained from 10^6 observations of the population mutant allele frequency trajectory.

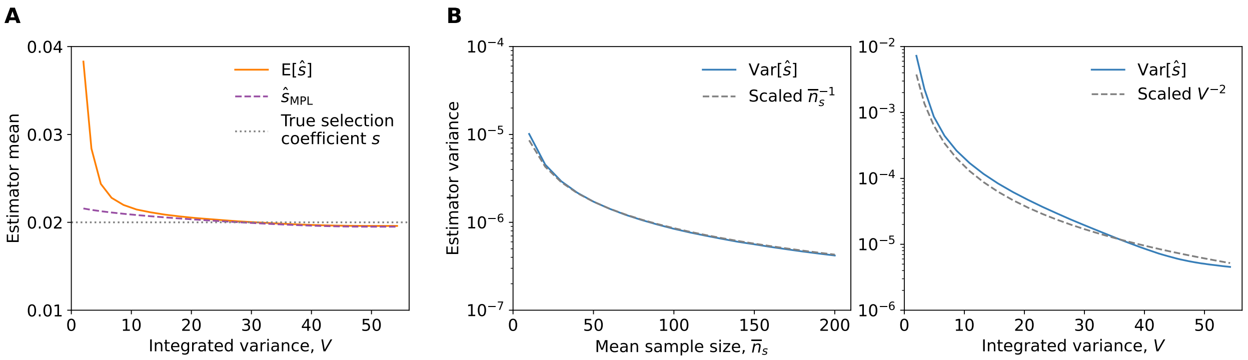

Figure S4 The estimator $\hat{s}$ (5) remains unbiased when the sample size is modeled as a time-varying Poisson random variable, with its variance scaling as $\mathcal{O}(\overline{n}_s^{-1}V^{-2})$. (A) shows the empirical estimator mean E[$\hat{s}$] with time-varying sample sizes drawn from Poisson distribution with a mean value of $\overline{n}_s = 20$, the estimate $\hat{s}_{\text{MPL}}$ (3) obtained from the population mutant allele frequency trajectory without limited sampling effect, along with the true selection coefficient s. (B) shows that the estimator variance Var[$\hat{s}$] is of order $\mathcal{O}(\overline{n}_s^{-1}V^{-2})$. In the *left* panel, we fixed the trajectory length $T = 450$ (thus fixed the integrated variance V) and plotted the empirical estimator variance with respect to the mean sample sizes $\overline{n}_s$ between 10 and 200. In the *right* panel, we fixed $\overline{n}_s = 20$ and plotted the empirical estimator variance with respect to integrated variance values V by varying T. Simulation parameters were selection coefficient $s = 0.02$, initial mutant allele frequency $x(0) = 0.1$, mutation probability $\mu = 10^{-3}$, with time sampling step $\Delta t = 10$. Empirical values of estimator mean and variance were obtained from 10^6 observations of the population mutant allele frequency trajectory simulated under the deterministic evolutionary model.

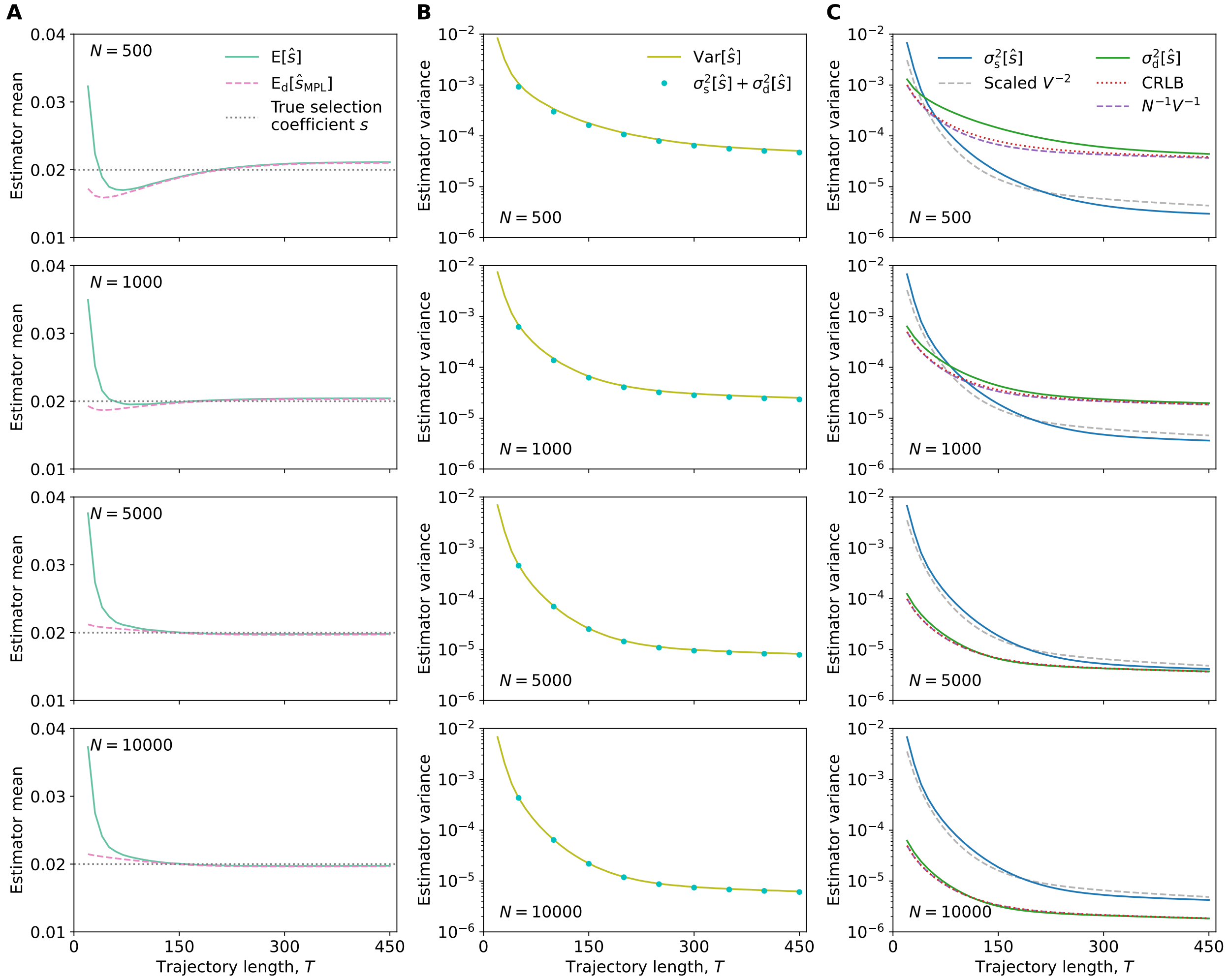

Figure S5 Derived analytical expressions for the estimator mean and variance accurately reflect the empirical results under the stochastic evolutionary model with various population sizes. (A) shows the empirical mean of $\hat{s}$ (5) under the joint effect of limited sampling and genetic drift, as well as the empirical mean of $\hat{s}_{\text{MPL}}$ (3) under genetic drift effect. (B) shows the empirical variance of $\hat{s}$ under the joint effect of limited sampling and genetic drift, as well as the empirical sum of sampling-only variance $\sigma_{\text{s}}^2[\hat{s}]$ and drift-only variance $\sigma_{\text{d}}^2[\hat{s}]$ when $T = (50, 100, \ldots, 450)$. (C) shows that the sampling-only variance $\sigma_{\text{s}}^2[\hat{s}]$ is of order $\mathcal{O}(V^{-2})$, while the drift-only variance $\sigma_{\text{d}}^2[\hat{s}]$ is approximately $1/NV$, close to its Cramér-Rao lower bound (CRLB). Empirical values of estimator mean and variance were obtained from 10^5 trajectories simulated over 450 generations. Simulation parameters were population size $N = (500, 1000, 2000, 5000, 10000)$, selection coefficient $s = 0.02$, initial mutant allele frequency $x(0) = 0.1$, mutation probability $\mu = 10^{-3}$, with sample size $n_s = 20$ and time sampling step $\Delta t = 10$.

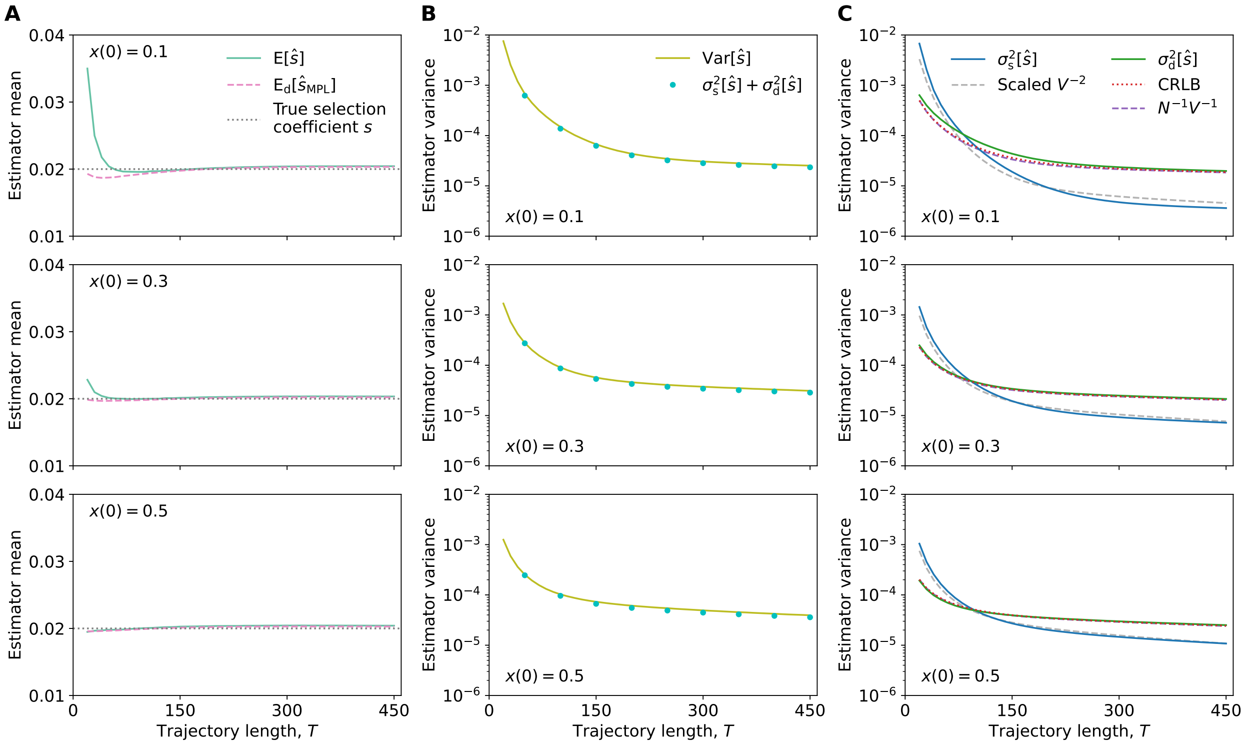

Figure S6 Derived analytical expressions for the estimator mean and variance accurately reflect the empirical results under the stochastic evolutionary model with various initial mutant allele frequencies. (A) shows the empirical mean of $\hat{s}$ (5) under the joint effect of limited sampling and genetic drift, as well as the empirical mean of $\hat{s}_{\mathrm{MPL}}$ (3) under genetic drift effect. (B) shows the empirical variance of $\hat{s}$ under the joint effect of limited sampling and genetic drift, as well as the empirical sum of sampling-only variance $\sigma_{\mathrm{s}}^2[\hat{s}]$ and drift-only variance $\sigma_{\mathrm{d}}^2[\hat{s}]$ when $T = (50, 100, \ldots, 450)$. (C) shows that the sampling-only variance $\sigma_{\mathrm{s}}^2[\hat{s}]$ is of order $\mathcal{O}(V^{-2})$, while the drift-only variance $\sigma_{\mathrm{d}}^2[\hat{s}]$ is approximately $1/NV$ (close to its CRLB). Empirical values of estimator mean and variance were obtained from 10^5 trajectories simulated over 450 generations. Simulation parameters were population size $N = 1000$, selection coefficient $s = 0.02$, initial mutant allele frequency $x(0) = (0.1, 0.3, 0.5)$, mutation probability $\mu = 10^{-3}$, with sample size $n_s = 20$ and time sampling step $\Delta t = 10$.

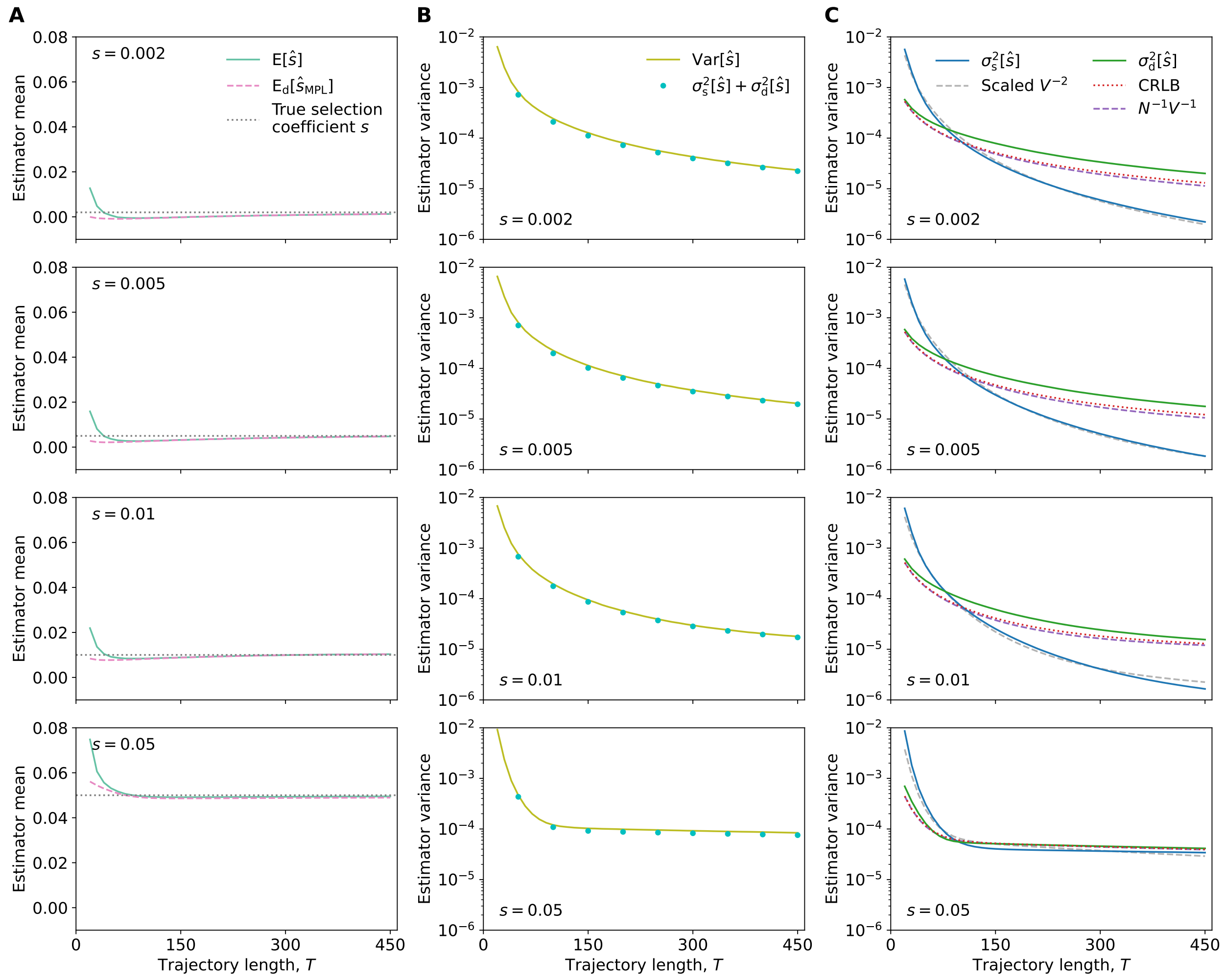

Figure S7 Derived analytical expressions for the estimator mean and variance accurately reflect the empirical results under the stochastic evolutionary model with various selection coefficients. (A) shows the empirical mean of $\hat{s}$ (5) under the joint effect of limited sampling and genetic drift, as well as the empirical mean of $\hat{s}_{\mathrm{MPL}}$ (3) under genetic drift effect. (B) shows the empirical variance of $\hat{s}$ under the joint effect of limited sampling and genetic drift, as well as the empirical sum of sampling-only variance $\sigma_s^2[\hat{s}]$ and drift-only variance $\sigma_d^2[\hat{s}]$ when $T = (50, 100, \ldots, 450)$. (C) shows that the sampling-only variance $\sigma_s^2[\hat{s}]$ is of order $\mathcal{O}(V^{-2})$, while the drift-only variance $\sigma_d^2[\hat{s}]$ is approximately $1/NV$ (close to its CRLB). Empirical values of estimator mean and variance were obtained from 10^5 trajectories simulated over 450 generations. Simulation parameters were population size $N = 1000$, selection coefficient $s = (0.002, 0.005, 0.01, 0.05)$, initial mutant allele frequency $x(0) = 0.1$, mutation probability $\mu = 10^{-3}$, with sample size $n_s = 20$ and time sampling step $\Delta t = 10$.

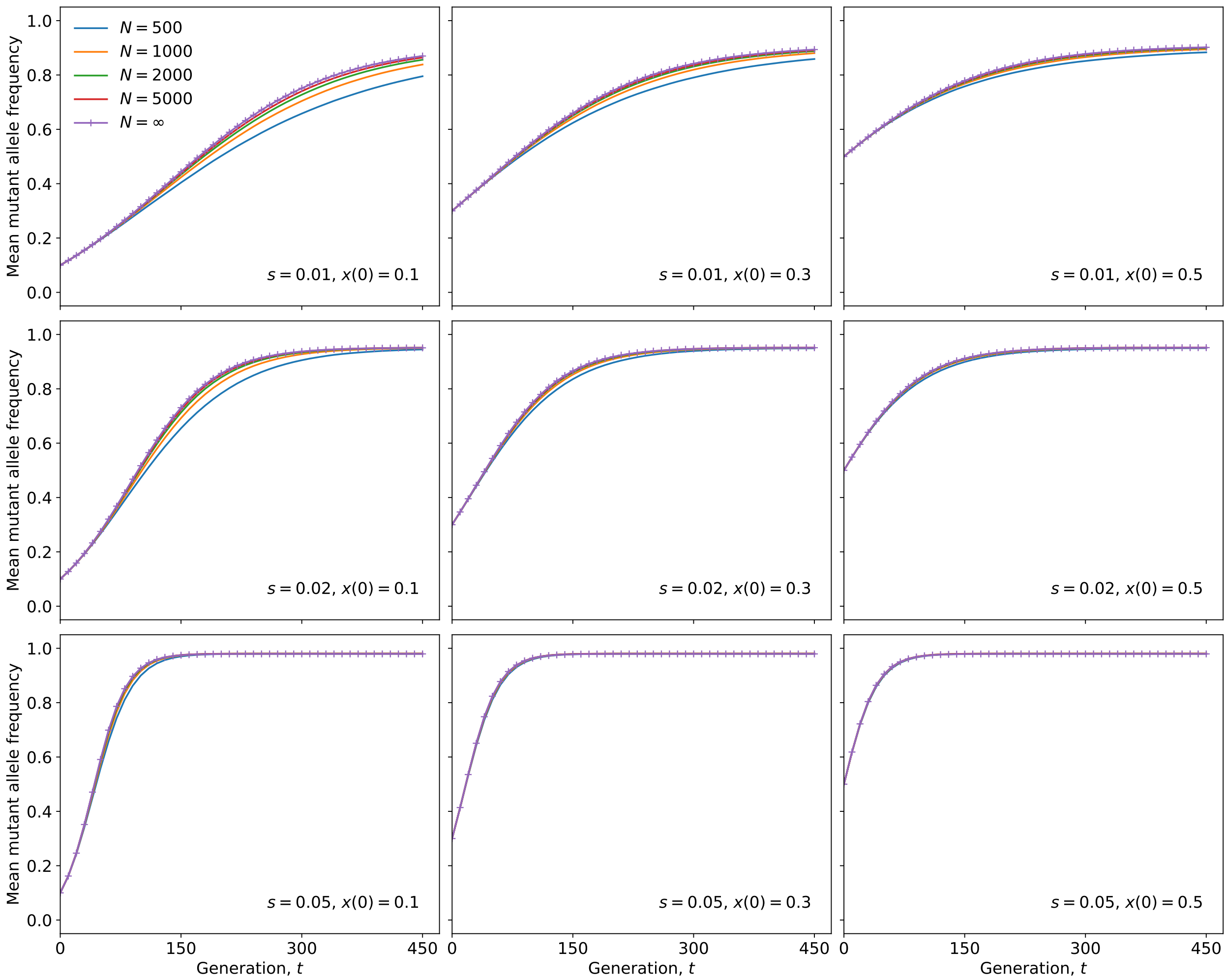

Figure S8 The mean trajectory over all simulated mutant allele frequency trajectories under the stochastic evolutionary model is effectively the trajectory under the deterministic evolutionary model. The population size was set to $N = (500, 1000, 2000, 5000)$ under the Wright-Fisher model to compare with the trajectory of the deterministic evolutionary model (i.e., infinite N). The selection coefficient was set to $s = (0.01, 0.02, 0.05)$ and the initial frequency was set to $x(0) = (0.1, 0.3, 0.5)$ as indicated. We simulated 10^5 trajectories for each setting over 450 generations with mutation probability $\mu = 10^{-3}$.

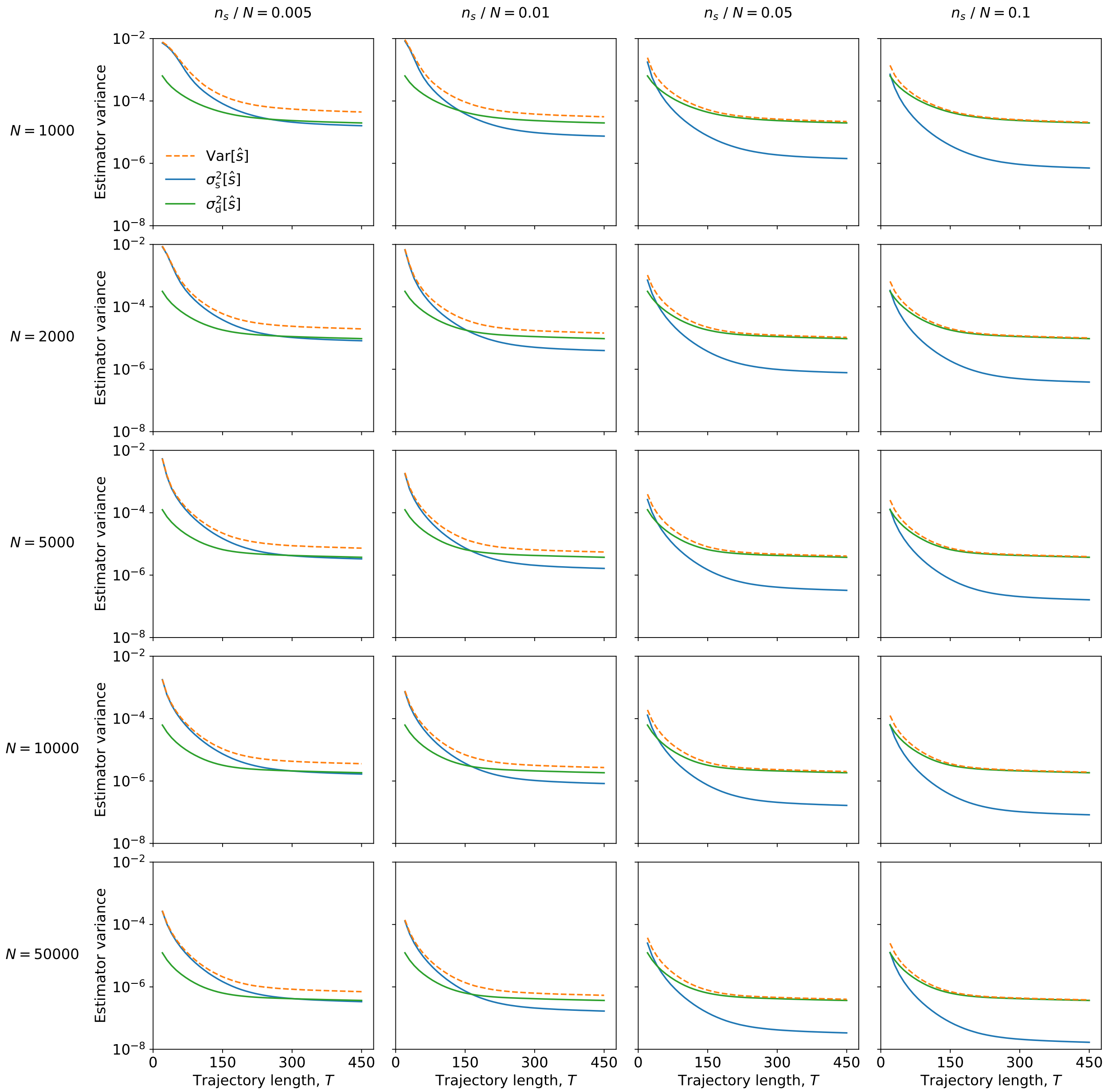

Figure S9 The drift-only variance $\sigma_{\mathrm{d}}^2[\hat{s}]$ dominates the sampling-only variance $\sigma_{\mathrm{s}}^2[\hat{s}]$ for longer trajectory length T (corresponding to larger integrated variance V). The estimator variance Var[$\hat{s}$] was empirically obtained from 10^5 trajectories under the joint effect of limited sampling and genetic drift. The sampling-only variance $\sigma_{\mathrm{s}}^2[\hat{s}]$ was empirically obtained from 10^6 observations of the mean trajectory over 10^5 population mutant allele frequencies simulated under the Wright-Fisher model. The drift-only variance $\sigma_{\mathrm{d}}^2[\hat{s}]$ was empirically obtained from these simulated 10^5 population mutant allele frequency trajectories. We simulated the trajectories over 450 generations under the Wright-Fisher model, with selection coefficient $s = 0.02$, initial mutant allele frequency $x(0) = 0.1$, and mutation probability $\mu = 10^{-3}$. The population size was set to $N = (1000, 2000, 5000, 10000, 50000)$ as indicated in each row, and sampling ratio was set to $n_s/N = (0.005, 0.01, 0.05, 0.1)$ as indicated in each column. The time sampling step was set to $\Delta t = 10$.

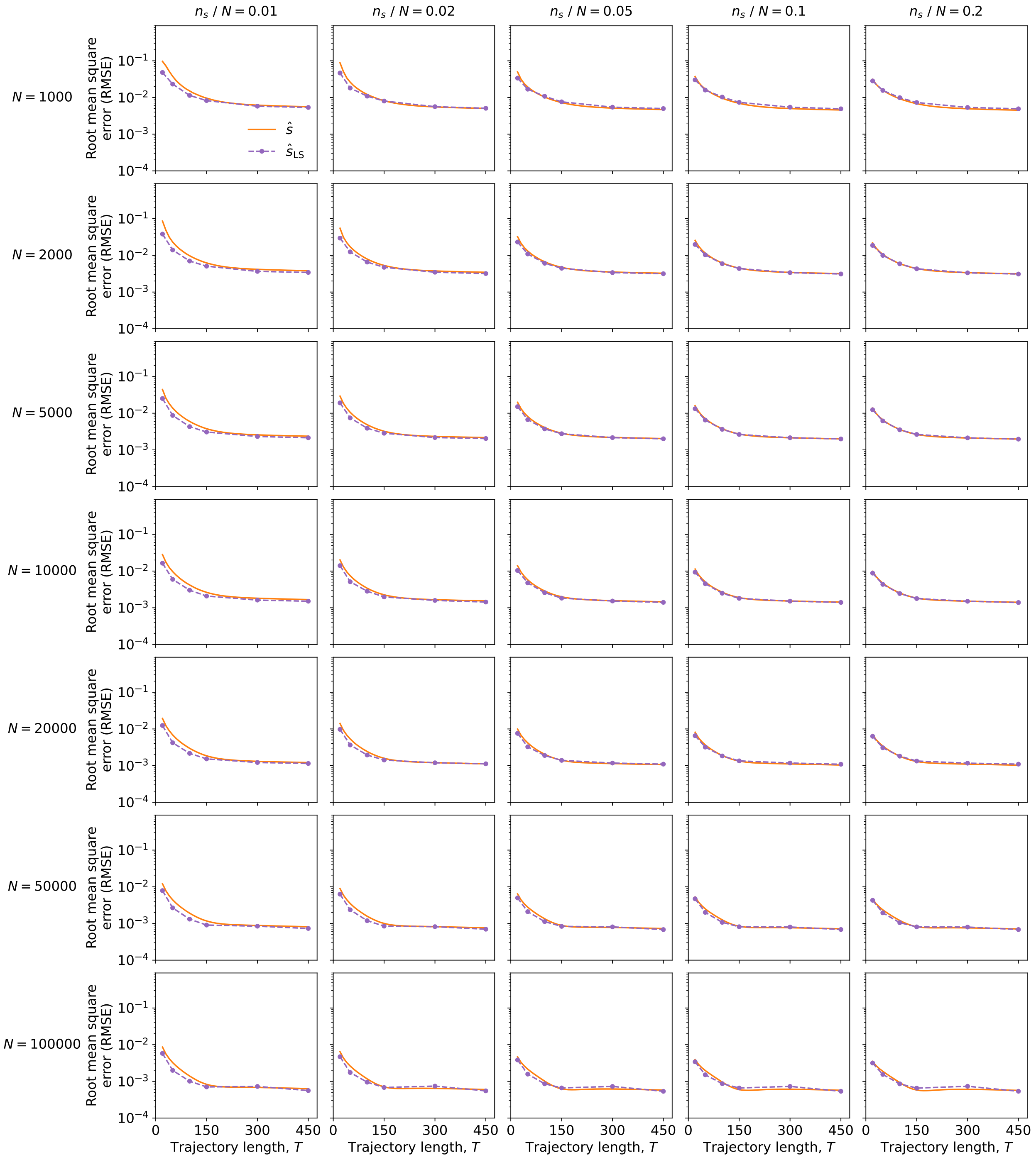

Figure S10 The estimators $\hat{s}$ and $\hat{s}_{\mathrm{LS}}$ demonstrate similar root mean square error (RMSE) performance under the joint effect of limited sampling and genetic drift. The RMSE of $\hat{s}$ was obtained from 10^5 observed mutant allele frequency trajectories, and the RMSE of $\hat{s}_{\mathrm{LS}}$ was obtained from 10^4 observed mutant allele frequency trajectories. We simulated the trajectories over 450 generations with selection coefficient $s = 0.02$, initial mutant allele frequency $x(0) = 0.1$, and mutation probability $\mu = 10^{-3}$. Population size was set to $N = (1000, 2000, 5000, 10000, 20000, 50000, 100000)$ and sampling ratio was set to $n_s/N = (0.01, 0.02, 0.05, 0.1, 0.2)$. The trajectories were observed with time sampling step $\Delta t = 10$.

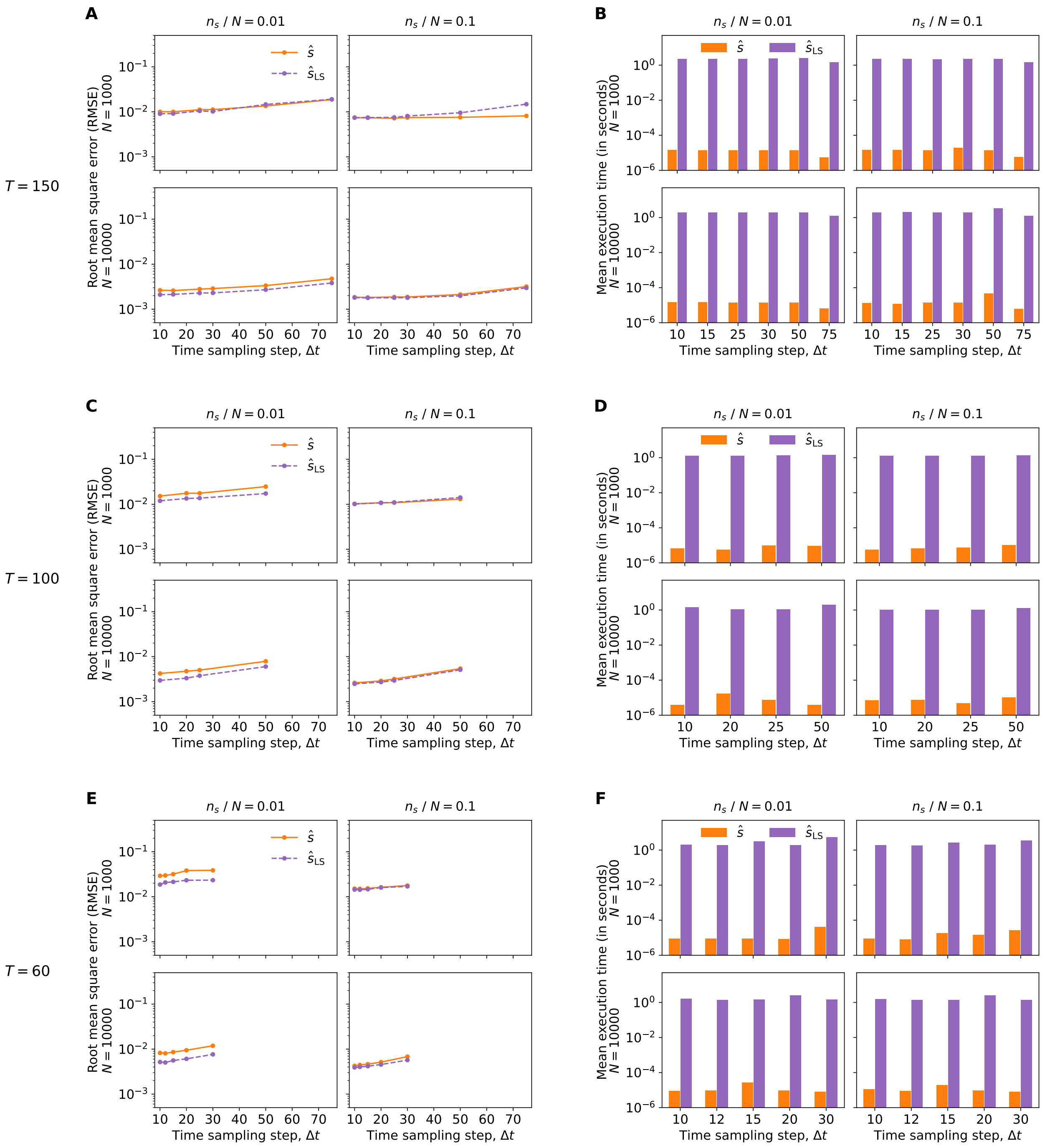

Figure S11 The root mean square error (RMSE) performance and execution time of estimators $\hat{s}$ and $\hat{s}_{\mathrm{LS}}$ with respect to time sampling step Δt. (A) shows the RMSE performance of $\hat{s}$ and $\hat{s}_{\mathrm{LS}}$ with trajectory length $T = 150$. (B) shows the mean execution time for $\hat{s}$ and $\hat{s}_{\mathrm{LS}}$ to estimate a selection coefficient from an observed mutant allele frequency trajectory with trajectory length $T = 150$. Similarly, (C) and (E) report RMSE for $T = (100, 60)$, respectively, while (D) and (F) report the corresponding mean execution times. Results were obtained from 1000 trajectories under the joint effect of limited sampling and genetic drift, with population and sample sizes as indicated. Simulation parameters were selection coefficient $s = 0.02$, initial mutant allele frequency $x(0) = 0.1$, and mutation probability $\mu = 10^{-3}$.

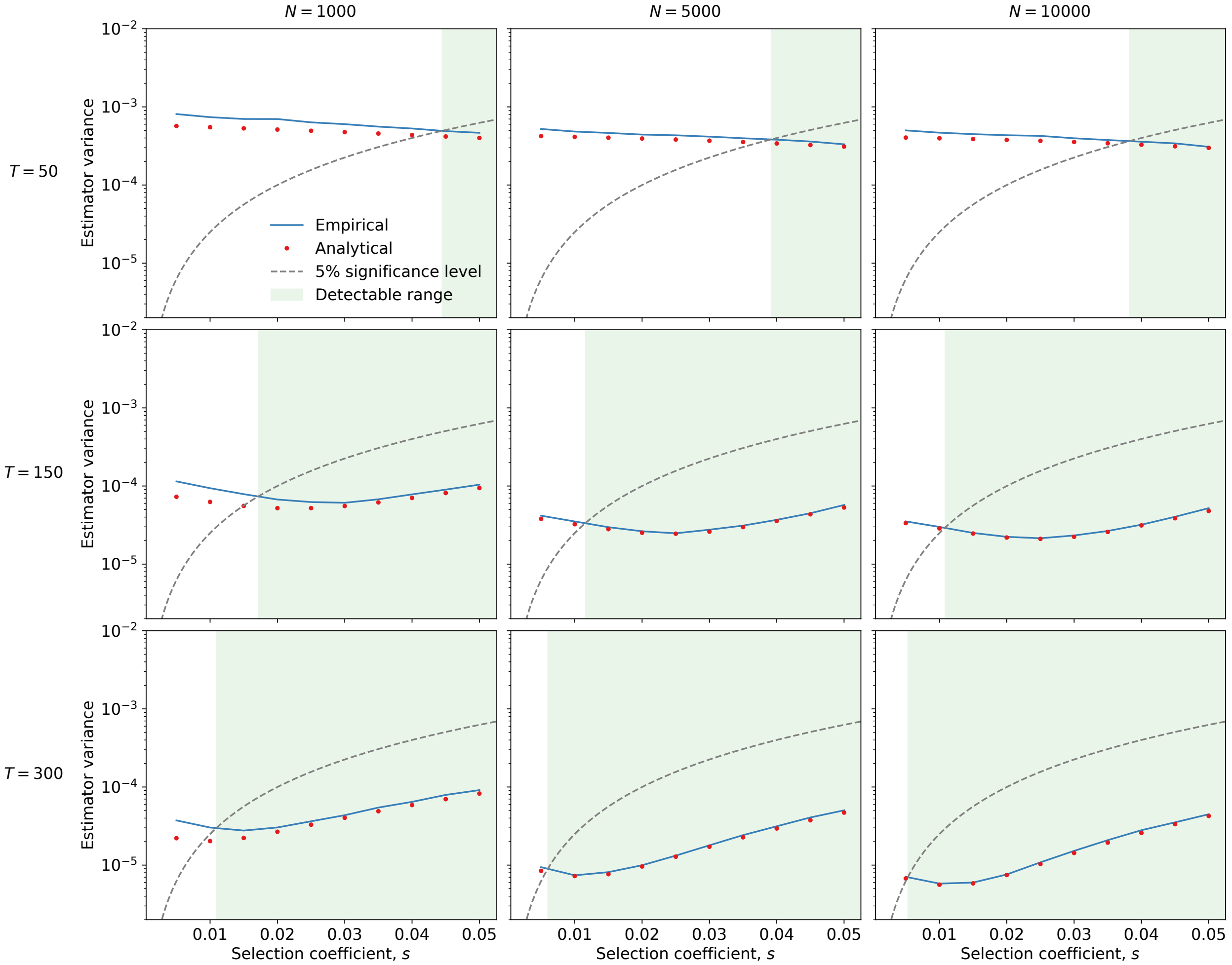

Figure S12 Analytical results of estimator variance Var[$\hat{s}$] reflect the empirical behavior and can be used to determine the range of detectable selection coefficients. Simulations were conducted with different values of population sizes N and trajectory lengths T. Empirical values of estimator variance were obtained from 10^4 trajectories under the joint effect of limited sampling and genetic drift. Analytical values of estimator variance were derived by summing the sampling-only variance and drift-only variance, as described in Materials and Methods. The dashed lines represent the threshold of estimator variance required to detect selection at the 5% significance level, assuming that the estimator $\hat{s}$ follows a Gaussian distribution. The shaded area indicates the range of selection coefficients detected by the estimator $\hat{s}$ at the 5% significance level, where the empirical variance of $\hat{s}$ is less than or equal to the threshold. Simulation parameters were initial mutant allele frequency $x(0) = 0.1$, mutation probability $\mu = 10^{-3}$, sample size $n_s = 20$, and time sampling step $\Delta t = 10$.

Supplementary Text

We begin by presenting the first four non-central moments of the observed mutant allele frequency $\hat{x}(t_k)$ under limited sampling effect. Subsequently, we derive the variance and covariance of the numerator and denominator in the expression of the MPL-based estimator $\hat{s}$ (25). These derivations are essential for analyzing the estimator variance Var[$\hat{s}$] due to limited sampling. Additionally, we demonstrate that the MPL-based estimator $\hat{s}$ is asymptotically Gaussian. We also derive the mean of estimator $\hat{s}_{\mathrm{MPL}}$ (23), as well as the second derivative of the log-likelihood in the MPL framework $\ell(s)$ with respect to s, which are required to study the mean and variance of $\hat{s}$ under the joint effect of limited sampling and genetic drift. To simplify the analysis and enhance interpretability, we assume a constant sample size n_s and a constant time sampling step Δt.

Moments of the observed mutant allele frequency $\hat{x}(t_k)$

We define the observed mutant allele frequency $\hat{x}(t_k)$ as the ratio of the mutant allele count $c(t_k)$, which is modeled as a binomial random variable, and the constant sample size n_s. For the ease of analysis in later sections, we derive the first four moments of $\hat{x}(t_k)$ from the moments of $c(t_k)$. The general expression of the non-central moments $\mathrm{E}\left[c^d(t_k)\right]$ with $d = (1, 2, \dots)$ is given by (see, for example, Knoblauch 2008)

$$\mathrm{E}\left[c^d(t_k)\right] = \sum_{j=0}^{d} \left\{ {d \atop j} \right\} n_s^{\underline{j}}\, x^j(t_k), \tag{S1}$$

where $\left\{ {d \atop j} \right\}$ denotes the Stirling numbers of the second kind, $n_s^{\underline{j}} = n_s(n_s-1)\dots(n_s-j+1)$ represents a falling factorial, and $x(t_k)$ is the population mutant allele frequency at generation t_k. Following (S1), we obtain the first-order moment

$$\mathrm{E}\left[\hat{x}(t_k)\right] = \mathrm{E}\left[\frac{c(t_k)}{n_s}\right] = x(t_k), \tag{S2}$$

the second-order moment

$$\mathrm{E}\left[\hat{x}^2(t_k)\right] = \mathrm{E}\left[\left(\frac{c(t_k)}{n_s}\right)^2\right] = \frac{1}{n_s}\left[(n_s-1)x^2(t_k) + x(t_k)\right] = x^2(t_k) + \frac{1}{n_s}v(t_k), \tag{S3}$$

where $v(t_k) = x(t_k)(1-x(t_k))$ is the population mutant allele variance at generation t_k, the third-order moment

$$\mathrm{E}\left[\hat{x}^3(t_k)\right] = \mathrm{E}\left[\left(\frac{c(t_k)}{n_s}\right)^3\right] = \frac{1}{n_s^2}\left[(n_s-1)(n_s-2)x^3(t_k) + 3(n_s-1)x^2(t_k) + x(t_k)\right], \tag{S4}$$

and the fourth-order moment

$$\mathrm{E}\left[\hat{x}^4(t_k)\right] = \mathrm{E}\left[\left(\frac{c(t_k)}{n_s}\right)^4\right] = \frac{1}{n_s^3}\left[(n_s-1)(n_s-2)(n_s-3)x^4(t_k) + 6(n_s-1)(n_s-2)x^3(t_k) + 7(n_s-1)x^2(t_k) + x(t_k)\right]. \tag{S5}$$

Correction factor to the observed mutant allele variance $\hat{v}(t_k)$

The observed mutant allele variance $\hat{v}(t_k)$ at generation t_k is defined as the product of the observed mutant allele frequency $\hat{x}(t_k)$ and the observed wild-type allele frequency $(1-\hat{x}(t_k))$ that are negatively correlated. Specifically, expectation of the observed mutant allele variance is expressed as

$$\begin{aligned}\mathrm{E}[\hat{v}(t_k)] &= \mathrm{E}\left[\hat{x}(t_k)\left(1-\hat{x}(t_k)\right)\right] \\ &= x(t_k) - \left(x^2(t_k) + \frac{1}{n_s}v(t_k)\right) \\ &= \left(1-\frac{1}{n_s}\right)v(t_k),\end{aligned} \tag{S6}$$

which differs from the population temporal mutant allele variance $v(t_k)$ by a factor of $\left(1-\frac{1}{n_s}\right)$. Thus, we multiply the inverse of this factor $\left(\frac{n_s}{n_s-1}\right)$ to each observed mutant allele variance in order to correct the bias due to limited sampling effect. This bias correction is applied to the estimator $\hat{s}$ expressed in (25).

Expectation of the numerator $\hat{D}$

We derive the expectation of the numerator $\hat{D}$ in (25), which represents the population mutant allele frequency change with corrections due to the mutation effect. Since $\hat{D}$ is a linear combination of the observed mutant allele frequencies $\hat{x}(t_k)$ at generations $(t_0, t_1, \ldots, t_K)$, we obtain

$$\begin{aligned}\mathrm{E}\left[\hat{D}\right] &= \mathrm{E}\left[\hat{x}(t_K) - \hat{x}(t_0) - \mu\Delta t\sum_{k=0}^{K-1}\left(1-2\hat{x}(t_k)\right)\right] \\ &= x(t_K) - x(t_0) - \mu\Delta t\sum_{k=0}^{K-1}\left(1-2x(t_k)\right),\end{aligned} \tag{S7}$$

which implies that $\hat{D}$ is an unbiased estimate of the true value computed from the population mutant allele frequency trajectory (i.e., without limited sampling effect). Expression of $\mathrm{E}\left[\hat{D}\right]$ is required for deriving the mean of $\hat{s}$ in (31).

Variance of the numerator $\hat{D}$

We derive the variance of $\hat{D}$ under sampling effect. Since $(\hat{x}(t_k))_{k=0}^{K}$ is considered as a set of independent random variables, the variance of its sum equals to the sum of its variance. Thus, term $\mathrm{Var}[\hat{D}]$ can be

decomposed to

$$\begin{aligned}\mathrm{Var}\left[\hat{D}\right] &= \mathrm{Var}\left[\hat{x}(t_K) - \hat{x}(t_0) - \mu\Delta t \sum_{k=0}^{K-1} (1 - 2\hat{x}(t_k))\right] \\ &= \mathrm{Var}\left[\hat{x}(t_K)\right] + (1 - 2\mu\Delta t)^2 \mathrm{Var}\left[\hat{x}(t_0)\right] + (2\mu\Delta t)^2 \sum_{k=1}^{K-1} \mathrm{Var}\left[\hat{x}(t_k)\right], \end{aligned} \tag{S8}$$

where the variance of the observed mutant allele frequency $\hat{x}(t_k)$ at generations $(t_0, t_1, \ldots, t_K)$ can be derived from its moments as

$$\mathrm{Var}\left[\hat{x}(t_k)\right] = \mathrm{E}\left[\hat{x}^2(t_k)\right] - \mathrm{E}^2\left[\hat{x}(t_k)\right] = \frac{1}{n_s} v(t_k). \tag{S9}$$

Therefore, Var[$\hat{D}$] is expressed as

$$\mathrm{Var}\left[\hat{D}\right] = \frac{1}{n_s}\left[v(t_K) + v(t_0) - 4\mu\Delta t v(t_0) + 4\mu^2\Delta t \left(\Delta t \sum_{k=0}^{K-1} v(t_k)\right)\right]. \tag{S10}$$

According to the definition of the integrated variance V (33), the above expression can be further simplified to

$$\mathrm{Var}\left[\hat{D}\right] = \frac{1}{n_s}\left[v(t_K) + v(t_0) - 4\mu\Delta t v(t_0) + 4\mu^2\Delta t V\right], \tag{S11}$$

which is required for deriving the variance of $\hat{s}$ in (34).

Expectation of the denominator $\hat{V}$

We derive the expectation of the denominator $\hat{V}$ in (25), which represents the observed integrated variance with correction due to limited sampling bias. As referred to in (25), term $\hat{V}$ is a linear combination of $\hat{v}(t_k)$ at generations $(t_0, t_1, \ldots, t_{K-1})$. Hence, its expectation is

$$\begin{aligned}\mathrm{E}[\hat{V}] &= \mathrm{E}\left[\left(\frac{n_s}{n_s - 1}\right)\Delta t \sum_{k=0}^{K-1} \hat{v}(t_k)\right] \\ &= \left(\frac{n_s}{n_s - 1}\right)\Delta t \sum_{k=0}^{K-1} \mathrm{E}[\hat{v}(t_k)]. \end{aligned} \tag{S12}$$

According to the expectation of the observed mutant allele variance given by (S6) and the integrated variance defined in (33), term E[$\hat{V}$] can be further simplified to

$$\begin{aligned}\mathrm{E}[\hat{V}] &= \left(\frac{n_s}{n_s - 1}\right)\Delta t \sum_{k=0}^{K-1} \left(1 - \frac{1}{n_s}\right) v(t_k) \\ &= \Delta t \sum_{k=0}^{K-1} v(t_k) \\ &= V, \end{aligned} \tag{S13}$$

which shows that the factor $\left(\frac{n_s}{n_s-1}\right)$ corrects the estimation bias of the observed mutant allele variances and consequently the time integration under sampling effect. Expression of $\mathrm{E}\left[\hat{V}\right]$ is required for deriving the mean of $\hat{s}$ in (31) and the variance of $\hat{s}$ in (34).

Variance of the denominator $\hat{V}$

We derive the variance of $\hat{V}$ under limited sampling effect. According to the definition of variance, $\mathrm{Var}[\hat{V}]$ expands to

$$\begin{aligned}\mathrm{Var}[\hat{V}] &= \mathrm{Var}\left[\left(\frac{n_s}{n_s-1}\right)\Delta t\sum_{k=0}^{K-1}\hat{v}(t_k)\right]\\ &= \left[\left(\frac{n_s}{n_s-1}\right)\Delta t\right]^2\sum_{k=0}^{K-1}\mathrm{Var}[\hat{v}(t_k)],\end{aligned}\tag{S14}$$

where $\mathrm{Var}[\hat{v}(t_k)]$ can be expressed in terms of the observed mutant allele frequencies $\hat{x}(t_k)$ as

$$\begin{aligned}\mathrm{Var}[\hat{v}(t_k)] &= \mathrm{Var}\left[\hat{x}(t_k)\left(1-\hat{x}(t_k)\right)\right]\\ &= \mathrm{Var}[\hat{x}(t_k)] + \mathrm{Var}[\hat{x}^2(t_k)] - 2\mathrm{Cov}[\hat{x}(t_k),\hat{x}^2(t_k)].\end{aligned}\tag{S15}$$

Based on the first four moments of $\hat{x}(t_k)$ derived above, $\mathrm{Var}[\hat{v}(t_k)]$ further expands to

$$\begin{aligned}\mathrm{Var}[\hat{v}(t_k)] &= \left(\mathrm{E}[\hat{x}^2(t_k)] - \mathrm{E}^2[\hat{x}(t_k)]\right) + \left(\mathrm{E}[\hat{x}^4(t_k)] - \mathrm{E}^2[\hat{x}^2(t_k)]\right) - 2\left(\mathrm{E}[\hat{x}^3(t_k)] - \mathrm{E}[\hat{x}(t_k)]\mathrm{E}[\hat{x}^2(t_k)]\right)\\ &= \frac{n_s-1}{n_s^3}\left[-2(2n_s-3)x^4(t_k) + 4(2n_s-3)x^3(t_k) - (5n_s-7)x^2(t_k) + (n_s-1)x(t_k)\right].\end{aligned}\tag{S16}$$

Taking (S14) and (S16) together with the definition of the integrated variance given by (33), $\mathrm{Var}[\hat{V}]$ can be expressed as

$$\begin{aligned}\mathrm{Var}[\hat{V}] &= \frac{\Delta t^2}{n_s}\sum_{k=0}^{K-1}\left[-4x^4(t_k) + 8x^3(t_k) - 5x^2(t_k) + x(t_k)\right] + \frac{2\Delta t^2}{n_s(n_s-1)}\sum_{k=0}^{K-1}\left[x^2(t_k)\left(1-x(t_k)\right)^2\right]\\ &= \frac{\Delta t}{n_s}\left[\left(\Delta t\sum_{k=0}^{K-1}v(t_k)\right) - \left(4 - \frac{2}{n_s-1}\right)\left(\Delta t\sum_{k=0}^{K-1}v^2(t_k)\right)\right]\\ &= \frac{\Delta t}{n_s}\left[V - \left(4 - \frac{2}{n_s-1}\right)\left(\Delta t\sum_{k=0}^{K-1}v^2(t_k)\right)\right].\end{aligned}\tag{S17}$$

Expression of $\mathrm{Var}[\hat{V}]$ is required for deriving the variance of $\hat{s}$ in (34).

Covariance between the numerator $\hat{D}$ *and the denominator* $\hat{V}$

We derive the covariance between the numerator $\hat{D}$ and the denominator $\hat{V}$ of the estimator $\hat{s}$. According to

the definition of $\hat{D}$ and $\hat{V}$ in (25), the term Cov[$\hat{D}$,$\hat{V}$] is expressed as

$$\mathrm{Cov}[\hat{D},\hat{V}] = \mathrm{Cov}\left[\left(\hat{x}(t_K) - \hat{x}(t_0) - \mu\Delta t \sum_{k=0}^{K-1} (1 - 2\hat{x}(t_k))\right), \left(\left(\frac{n_s}{n_s - 1}\right) \Delta t \sum_{k=0}^{K-1} \hat{v}(t_k)\right)\right]. \tag{S18}$$

Based on the linear property of covariance, the above expression can be decomposed to

$$\begin{aligned}
\mathrm{Cov}[\hat{D},\hat{V}] &= \left(\frac{n_s}{n_s - 1}\right) \Delta t \,\mathrm{Cov}\left[\left(\hat{x}(t_K) - \hat{x}(t_0) + 2\mu\Delta t \sum_{k=0}^{K-1} \hat{x}(t_k)\right), \left(\sum_{k=0}^{K-1} \hat{x}(t_k) - \sum_{k=0}^{K-1} \hat{x}^2(t_k)\right)\right] \\
&= \left(\frac{n_s}{n_s - 1}\right) \Delta t \left\{\mathrm{Cov}\left[\hat{x}(t_K), \sum_{k=0}^{K-1} \hat{x}(t_k)\right] - \mathrm{Cov}\left[\hat{x}(t_K), \sum_{k=0}^{K-1} \hat{x}^2(t_k)\right]\right. \\
&\qquad - \mathrm{Cov}\left[\hat{x}(t_0), \sum_{k=0}^{K-1} \hat{x}(t_k)\right] + \mathrm{Cov}\left[\hat{x}(t_0), \sum_{k=0}^{K-1} \hat{x}^2(t_k)\right] \\
&\qquad \left. + 2\mu\Delta t\, \mathrm{Var}\left[\sum_{k=0}^{K-1} \hat{x}(t_k)\right] - 2\mu\Delta t\, \mathrm{Cov}\left[\sum_{k=0}^{K-1} \hat{x}(t_k), \sum_{k=0}^{K-1} \hat{x}^2(t_k)\right]\right\}.
\end{aligned} \tag{S19}$$

Since the sampling process is assumed to be independent, $(\hat{x}(t_k))_{k=0}^{K}$ is a set of independent random variables that result in zero covariance between $\hat{x}(t_k)$ and $\sum_{k=0}^{K-1} \hat{x}(t_k)$, and between $\hat{x}(t_k)$ and $\sum_{k=0}^{K-1} \hat{x}^2(t_k)$. Therefore, the first two decomposed covariance terms in (S19), i.e., $\mathrm{Cov}[\hat{x}(t_K), \sum_{k=0}^{K-1} \hat{x}(t_k)]$, and $\mathrm{Cov}[\hat{x}(t_K), \sum_{k=0}^{K-1} \hat{x}^2(t_k)]$, equal to zero. Using this independence property and the derived moments of $\hat{x}(t_k)$, the covariance between $\hat{x}(t_0)$ and $\sum_{k=0}^{K-1} \hat{x}(t_k)$ can be simplified as

$$\begin{aligned}
\mathrm{Cov}\left[\hat{x}(t_0), \sum_{k=0}^{K-1} \hat{x}(t_k)\right] &= \mathrm{Var}[\hat{x}(t_0)] \\
&= \frac{1}{n_s} v(t_0).
\end{aligned} \tag{S20}$$

The covariance between $\hat{x}(t_0)$ and $\sum_{k=0}^{K-1} \hat{x}^2(t_k)$, which is the fourth term of (S19), can be simplified as

$$\begin{aligned}
\mathrm{Cov}\left[\hat{x}(t_0), \sum_{k=0}^{K-1} \hat{x}^2(t_k)\right] &= \mathrm{Cov}[\hat{x}(t_0), \hat{x}^2(t_0)] \\
&= \mathrm{E}[\hat{x}^3(t_0)] - \mathrm{E}[\hat{x}(t_0)]\mathrm{E}[\hat{x}^2(t_0)] \\
&= \frac{1}{n_s^2} v(t_0) \left(1 - 2x(t_0) + 2n_s x(t_0)\right).
\end{aligned} \tag{S21}$$

The fifth term of (S19) is the variance of $\sum_{k=0}^{K-1} \hat{x}(t_k)$, and is given by

$$\mathrm{Var}\left[\sum_{k=0}^{K-1} \hat{x}(t_k)\right] = \frac{1}{n_s} \sum_{k=0}^{K-1} v(t_k), \tag{S22}$$

while the last term in (S19) is the covariance between $\sum_{k=0}^{K-1}\hat{x}(t_k)$ and $\sum_{k=0}^{K-1}\hat{x}^2(t_k)$, and is given by

$$\begin{aligned}\mathrm{Cov}\left[\sum_{k=0}^{K-1}\hat{x}(t_k),\sum_{k=0}^{K-1}\hat{x}^2(t_k)\right]&=\sum_{k=0}^{K-1}\mathrm{Cov}\left[\hat{x}(t_k),\hat{x}^2(t_k)\right]\\&=\frac{1}{n_s^2}\sum_{k=0}^{K-1}v(t_k)\left(1-2x(t_k)+2n_s x(t_k)\right).\end{aligned}\tag{S23}$$

Lastly, substituting the results of the six decomposed terms to (S19) simplifies Cov$[\hat{D},\hat{V}]$ to

$$\mathrm{Cov}[\hat{D},\hat{V}]=-\frac{\Delta t}{n_s}\left[v(t_0)\left(1-2x(t_0)\right)-2\mu\Delta t\sum_{k=0}^{K-1}v(t_k)\left(1-2x(t_k)\right)\right].\tag{S24}$$

Expression of $\mathrm{Cov}[\hat{D},\hat{V}]$ is required for deriving the variance of $\hat{s}$ in (34).

Asymptotic distribution of $\hat{V}$

The term $\hat{V}$ in (25) can be taken as the sum of K independently observed mutant allele variances at generations $(t_0,t_1,...t_{K-1})$, multiplied by the constant time sampling step Δt and the bias correction factor indicated earlier. Here we demonstrate its asymptotic Gaussianity using the central limit theorem as K approaches infinity (i.e., with the trajectory length T). We conduct the proof by first showing that the sum of observed mutant allele variances, $\sum_{k=0}^{K-1}\hat{v}(t_k)$, converges in distribution to a Gaussian random variable and then relating this result to $\hat{V}$. For notational convenience, we define the sum of the limited sampling variance of each observed mutant allele variance $\hat{v}(t_k)$ at generations $(t_0,t_1,\ldots,t_{K-1})$ as s_K^2, i.e.,

$$s_K^2=\sum_{k=0}^{K-1}\mathrm{Var}\left[\hat{v}(t_k)\right].\tag{S25}$$

We assumed a sufficiently large mutation probability, i.e., $\mu=\mathcal{O}(N^{-1})$, such that mutant alleles are continuously generated within the population, keeping the locus polymorphic and the population mutant allele frequency trajectory away from the boundaries (i.e., zero and one). Thus, the variance sum s_K^2 approaches infinity as $K\to\infty$. Since the observed mutant allele frequency $\hat{x}(t_k)$ was assumed to be bounded on a closed interval $[0,1]$ at generations $(t_0,t_1,...t_{K-1})$, the observed mutant allele variance $\hat{v}(t_k)=\hat{x}(t_k)(1-\hat{x}(t_k))$ is also bounded. Therefore, the centralized random variable $\hat{w}(t_k)=\hat{v}(t_k)-\mathrm{E}[\hat{v}(t_k)]$ is bounded, which follows $|\hat{w}(t_k)|<1$ and gives

$$\mathrm{E}\left[|\hat{w}(t_k)|^3\right]<\mathrm{E}\left[|\hat{w}(t_k)|^2\right],\tag{S26}$$

implying that for $\delta = 1$,

$$\lim_{K\to\infty} \frac{1}{s_K^{2+\delta}} \sum_{k=0}^{K-1} \mathrm{E}\left[|\hat{w}(t_k)|^{2+\delta}\right] < \lim_{K\to\infty} \frac{1}{s_K^3} \sum_{k=0}^{K-1} \mathrm{E}\left[|\hat{w}(t_k)|^2\right]. \tag{S27}$$

Since $\hat{w}(t_k)$ is zero-mean by its definition, and the difference between $\hat{w}(t_k)$ and $\hat{v}(t_k)$ is a constant, i.e., $\mathrm{E}[\hat{v}(t_k)]$,

$$\mathrm{E}\left[|\hat{w}(t_k)|^2\right] = \mathrm{Var}\left[\hat{w}(t_k)\right] = \mathrm{Var}\left[\hat{v}(t_k)\right], \tag{S28}$$

which implies that

$$\sum_{k=0}^{K-1} \mathrm{E}\left[|\hat{w}(t_k)|^2\right] = \sum_{k=0}^{K-1} \mathrm{Var}\left[\hat{v}(t_k)\right] = s_K^2. \tag{S29}$$

Substituting (S29) to (S27) gives

$$\lim_{K\to\infty} \frac{1}{s_K^{2+\delta}} \sum_{k=0}^{K-1} \mathrm{E}\left[|\hat{w}(t_k)|^{2+\delta}\right] < \lim_{K\to\infty} \frac{1}{s_K} = 0 \tag{S30}$$

for $\delta = 1$, which satisfies the condition established by Lyapunov (see, for example, Theorem 27.3 in Billingsley 1995). Therefore, the Lyapunov central limit theorem implies that $\sum_{k=0}^{K-1} \hat{v}(t_k)$ converges in distribution to a Gaussian random variable as the number of time points K approaches infinity. Since $\hat{V}$ is defined as $\sum_{k=0}^{K-1} \hat{v}(t_k)$ multiplied by a constant $\Delta t \left(\frac{n_s}{n_s-1}\right)$, the convergence property also applies to $\hat{V}$.

Coefficient of variation of $\hat{V}$

The coefficient of variation is defined as the ratio of the standard deviation to the mean. For the observed integrated variance $\hat{V}$ in (25), its coefficient of variation can be written as

$$\mathrm{C_v}(\hat{V}) = \sqrt{\frac{\mathrm{Var}[\hat{V}]}{V^2}}. \tag{S31}$$

Using (S17), we obtain

$$\begin{aligned}\frac{\mathrm{Var}[\hat{V}]}{V^2} &= \frac{\Delta t}{n_s V^2}\left[V - \left(4 - \frac{2}{n_s - 1}\right)\Delta t \sum_{k=0}^{K-1} v^2(t_k)\right] \\ &= \frac{\Delta t}{n_s V}\left[1 - \left(4 - \frac{2}{n_s - 1}\right)\frac{\Delta t \sum_{k=0}^{K-1} v^2(t_k)}{V}\right] \\ &= \frac{\Delta t}{n_s V}\left[1 - \left(4 - \frac{2}{n_s - 1}\right)\zeta\right],\end{aligned} \tag{S32}$$

where we have rearranged the factor of integrated variance V and defined

$$\zeta := \frac{\Delta t \sum_{k=0}^{K-1} v^2(t_k)}{V} \tag{S33}$$

for the ease of analysis. Here the denominator represents the population mutant allele variance integrated along the trajectory, as defined in (33), while the numerator represents the square of population mutant allele variance integrated along the trajectory.

The term ζ is bounded on a closed interval, although its denominator and the numerator in (S33) increase with the trajectory length T. Since the population mutant allele variances follow $(v(t_k))_{k=0}^{K-1} \leq v_{\max}$, where $v_{\max}$ is the maximum value of the set, we obtain

$$\Delta t \sum_{k=0}^{K-1} v^2(t_k) \leq v_{\max}\, \Delta t \sum_{k=0}^{K-1} v(t_k) = v_{\max} V, \tag{S34}$$

where the equality holds only when the frequency trajectory $(x(t_k))_{k=0}^{K-1}$ remains constant for all k. According to the Cauchy-Schwarz inequality (see, for example, 8.1 in Sedrakyan and Sedrakyan 2018),

$$\sum_{k=0}^{K-1} v^2(t_k) \geq \frac{1}{K} \left(\sum_{k=0}^{K-1} v(t_k) \right)^2, \tag{S35}$$

which implies that $\Delta t \sum_{k=0}^{K-1} v^2(t_k)$ follows

$$\Delta t \sum_{k=0}^{K-1} v^2(t_k) \geq \left(\frac{1}{K} \sum_{k=0}^{K-1} v(t_k) \right) \left(\Delta t \sum_{k=0}^{K-1} v(t_k) \right) = v_{\mathrm{avg}} V, \tag{S36}$$

where $v_{\mathrm{avg}} = \frac{1}{K} \sum_{k=0}^{K-1} v(t_k)$ denotes the average population mutant allele variance over all K generations $(t_0, t_1, \ldots, t_{K-1})$. Taking (S34) and (S36) together gives

$$v_{\mathrm{avg}} V \leq \Delta t \sum_{k=0}^{K-1} v^2(t_k) \leq v_{\max} V. \tag{S37}$$

According to (S33), we obtain

$$\zeta \in [v_{\mathrm{avg}}, v_{\max}], \tag{S38}$$

which gives the upper and lower bounds of ζ appeared in (S32). As the population mutant allele frequency is bounded on $(0, 1)$ and the population mutant allele variance is bounded on $(0, 0.25]$, both v_{avg} and $v_{\max}$ are bounded on a half-open interval $(0, 0.25]$. Therefore, the ratio ζ is bounded on $(0, 0.25]$. Because the product of $(4 - 2/(n_s - 1))$ and ζ is always greater than zero and less than one, the sum of components within the square bracket of (S32) is bounded on $(0, 1)$ for any $n_s > 1$. Thus, the coefficient of variation $\mathrm{C_v}(\hat{V})$ follows

$$0 < \mathrm{C_v}(\hat{V}) < \sqrt{\frac{\Delta t}{n_s V}}, \tag{S39}$$

where its upper bound $\sqrt{\Delta t/(n_s V)}$ approaches zero as either the sample size n_s or the integrated variance V approaches infinity. Given the non-negative $C_v(\hat{V})$, the coefficient of variation of $\hat{V}$ approaches zero as n_s or V approaches infinity.

Mean of the estimator $\hat{s}_{MPL}$ under genetic drift effect

The estimator $\hat{s}_{\text{MPL}}$ (23) is derived under the MPL framework, which considers genetic drift effect but ignores limited sampling effect. We assume constant time sampling step Δt in the following analysis for the mean of $\hat{s}_{\text{MPL}}$, however this assumption can be readily relaxed. The estimator $\hat{s}_{\text{MPL}}$ is expressed as

$$\hat{s}_{\text{MPL}} = \frac{x(t_K) - x(t_0) - \mu\Delta t \sum_{k=0}^{K-1}(1-2x(t_k))}{\Delta t \sum_{k=0}^{K-1} v(t_k)} = \frac{D}{V}, \tag{S40}$$

where the numerator is denoted as D and the denominator is denoted as V for notational convenience.

To obtain an analytical approximation for the mean of $\hat{s}_{\text{MPL}}$, we apply multivariate Taylor series expansion with respect to D and V expanded about their expectations $\text{E}[D]$ and $\text{E}[V]$. The first-order expansion gives the estimator mean as

$$\text{E}_\text{d}[\hat{s}_{\text{MPL}}] = \text{E}_\text{d}\left[\frac{D}{V}\right] = \frac{\text{E}_\text{d}[D]}{\text{E}_\text{d}[V]}, \tag{S41}$$

where E_d represents the mean over trajectories under genetic drift effect. Given the initial mutant allele frequency $x(t_0)$, the expectation of D in (S41) can be expressed as

$$\begin{aligned}
\text{E}_\text{d}[D] &= \text{E}_\text{d}\left[x(t_K) - x(t_0) - \mu\Delta t \sum_{k=0}^{K-1}(1-2x(t_k)) \middle| x(t_0)\right] \\
&= \sum_{k=0}^{K-1} \text{E}_\text{d}\left[x(t_{k+1}) - x(t_k) - \mu\Delta t\,(1-2x(t_k)) | x(t_0)\right] \\
&= \sum_{k=0}^{K-1} \text{E}_\text{d}\left[\text{E}_\text{d}\left[x(t_{k+1}) - x(t_k) - \mu\Delta t\,(1-2x(t_k)) | x(t_k)\right] | x(t_0)\right].
\end{aligned} \tag{S42}$$

According to the law of total expectation, the expected frequency of mutant allele at generation t_{k+1} under genetic drift effect, conditioned on the frequency $x(t_k)$ at generation t_k, admits

$$\begin{aligned}
\text{E}_\text{d}\left[x(t_{k+1}) | x(t_k)\right] &= \text{E}_\text{d}\left[x(t_k + \Delta t) | x(t_k)\right] \\
&= \text{E}_\text{d}\left[\text{E}_\text{d}\left[x(t_k + \Delta t) | x(t_k + \Delta t - 1)\right] | x(t_k)\right].
\end{aligned} \tag{S43}$$

Under the single-locus WF model and the assumption that both the selection coefficient s and the mutation probability μ are of order $\mathcal{O}(N^{-1})$, i.e., a parameter regime that is consistent with the single-locus MPL

framework, we obtain

$$\begin{aligned}\mathrm{E_d}\left[x(t_k+\Delta t)|x(t_k+\Delta t-1)\right] &= \frac{(1+s)\ x(t_k+\Delta t-1)+\mu[(1-x(t_k+\Delta t-1))-(1+s)\ x(t_k+\Delta t-1)]}{1+s\ x(t_k+\Delta t-1)} \\ &= x(t_k+\Delta t-1)+s\ v(t_k+\Delta t-1)+\mu(1-2x(t_k+\Delta t-1))+\mathcal{O}(N^{-2}),\end{aligned} \tag{S44}$$

where $v(t_k+\Delta t-1)$ is the mutant allele variance at generation $(t_k+\Delta t-1)$ that follows

$$v(t_k+\Delta t-1) = x(t_k+\Delta t-1)\ (1-x(t_k+\Delta t-1)). \tag{S45}$$

By iterating the above procedure Δt times, we have

$$\mathrm{E_d}\left[x(t_k+\Delta t)|x(t_k)\right] = x(t_k)+s\sum_{\tau=0}^{\Delta t-1}\mathrm{E}[v(t_k+\tau)|x(t_k)]+\mu\Delta t(1-2x(t_k))+\mathcal{O}(N^{-2}), \tag{S46}$$

where $\mathrm{E}[v(t_k+\tau)|x(t_k)]$ follows

$$\mathrm{E}[v(t_k+\tau)|x(t_k)] = v(t_k)+\mathcal{O}(N^{-1}), \tag{S47}$$

for $\tau=(0,1,\ldots,\Delta t-1)$. Substituting the above expression to (S46), we obtain

$$\mathrm{E_d}\left[x(t_k+\Delta t)|x(t_k)\right] = x(t_k)+\Delta t\left(sv(t_k)+\mu(1-2x(t_k))\right)+\mathcal{O}(N^{-2}). \tag{S48}$$

Approximating the frequency change up to the order of $\mathcal{O}(N^{-1})$ and rearranging the above expression then gives

$$\mathrm{E_d}\left[x(t_k+\Delta t)-x(t_k)-\mu\Delta t(1-2x(t_k))|x(t_k)\right] \approx s\Delta t v(t_k), \tag{S49}$$

with generation $t_k+\Delta t=t_{k+1}$. Thus, substituting the above expression to (S42), we obtain the mean of D as

$$\mathrm{E_d}[D] = s\ \Delta t\sum_{k=0}^{K-1}\mathrm{E_d}\left[v(t_k)|x(t_0)\right]. \tag{S50}$$

As defined in (S40), the mean of V under genetic drift effect, given the initial mutant allele frequency $x(t_0)$, can be written as

$$\mathrm{E_d}[V] = \mathrm{E_d}\left[\Delta t\sum_{k=0}^{K-1}v(t_k)\middle|x(t_0)\right] = \Delta t\sum_{k=0}^{K-1}\mathrm{E_d}[v(t_k)|x(t_0)], \tag{S51}$$

which has a very similar form to $\mathrm{E_d}[D]$ as given in (S50). Substituting the results in (S50) and (S51) to (S41),

we finally obtain

$$\mathrm{E}_{\mathrm{d}}[\hat{s}_{\mathrm{MPL}}] = \frac{\mathrm{E}_{\mathrm{d}}[D]}{\mathrm{E}_{\mathrm{d}}[V]} = \frac{s\ \Delta t \sum_{k=0}^{K-1} \mathrm{E}_{\mathrm{d}}\left[v(t_k)|x(t_0)\right]}{\Delta t \sum_{k=0}^{K-1} \mathrm{E}_{\mathrm{d}}[v(t_k)|x(t_0)]} = s, \tag{S52}$$

which indicates that the mean of estimator $\hat{s}_{\mathrm{MPL}}$ under the genetic drift effect is effectively the true selection coefficient s.

Second derivative of the log-likelihood $\ell(s)$ with respect to the selection coefficient s

We denote $\ell(s)$ as the log-likelihood of the selection coefficient s given a population mutant allele frequency trajectory $(x(t_k))_{k=1}^{K}$ with a fixed initial frequency $x(t_0)$, a finite population size N, and a constant mutation probability μ. i.e.,

$$\ell(s) := \log \mathcal{L}(s|\ (x(t_k))_{k=0}^{K}, N, \mu) = \log \left(P\left((x(t_k))_{k=1}^{K} | x(t_0), N, \mu, s \right) \right). \tag{S53}$$

In the MPL framework, the WF model is approximated by a diffusion process, and the transition probability density of the frequency change is approximated with a path integral. Under these approximations, the probability $P\left((x(t_k))_{k=1}^{K}|x(t_0), N, \mu, s\right)$ in (S53) has the following Gaussian form:

$$P\left((x(t_k))_{k=1}^{K} | x(t_0), N, \mu, s \right) \approx \left(\prod_{k=0}^{K-1} \sqrt{\frac{N}{2\pi \Delta t v(t_k)}} \mathrm{d}x(t_{k+1}) \right) \exp\left(-\frac{N}{2} S\left((x(t_k))_{k=0}^{K} \right) \right), \tag{S54}$$

where $\mathrm{d}x(t_{k+1})$ is a constant that denotes the small frequency difference accounting for the quantization of continuous frequency space, $v(t_k) = x(t_k)(1 - x(t_k))$ is the population mutant allele variance at generation t_k, and $S\left((x(t_k))_{k=0}^{K}\right)$ is expressed as

$$S\left((x(t_k))_{k=0}^{K} \right) = \sum_{k=0}^{K-1} \frac{1}{\Delta t v(t_k)} \left[x(t_{k+1}) - x(t_k) - \Delta t \left(s v(t_k) + \mu(1 - 2x(t_k)) \right) \right]^2. \tag{S55}$$

After dropping terms independent of s, we obtain the second derivative of $\ell(s)$ with respect to s as

$$\begin{aligned} \frac{\partial^2}{\partial s^2} \ell(s) &= -\frac{N}{2} \frac{\partial^2}{\partial s^2} S\left((x(t_k))_{k=0}^{K} \right) \\ &= -\frac{N}{2} \sum_{k=0}^{K-1} \frac{1}{\Delta t v(t_k)} \frac{\partial^2}{\partial s^2} \left[x(t_{k+1}) - x(t_k) - \Delta t \left(s v(t_k) + \mu(1 - 2x(t_k)) \right) \right]^2 \\ &= -N\Delta t \sum_{k=0}^{K-1} v(t_k), \end{aligned} \tag{S56}$$

where $\Delta t \sum_{k=0}^{K-1} v(t_k)$ is the integrated variance of the population mutant allele frequency trajectory $(x(t_k))_{k=0}^{K}$ as defined in (33). Thus, the second derivative of $\ell(s)$ with respect to the selection coefficient s can be written as the negative product of the population size and the integrated variance.

Literature cited

Billingsley P. 1995. *Probability and Measure*. John Wiley & Sons. Third edition.

Knoblauch A. 2008. Closed-form expressions for the moments of the binomial probability distribution. SIAM Journal on Applied Mathematics. 69:197–204.

Sedrakyan H, Sedrakyan N. 2018. *Algebraic Inequalities*. Springer International Publishing.
